# Joint validation of yield and inter-annual consistency and elite lines selection in 52 advanced wheat lines based on a PCA-membership function-TOPSIS collaborative system

**DOI:** 10.3389/fpls.2026.1907156

**Published:** 2026-08-25

**Authors:** Yinghao Zhang, Mingjun Ai, Shuaiguo Ma, Fangyuan Xu, Qi Li, Hongna Guo, Yin-Gang Hu, Qian Liu, Qinglin Wen

**Affiliations:** 1 College of Agriculture, Tarim University, Alar, China; 2 Key Laboratory of Genetic Improvement and Efficient Production for Specialty Crops in Arid Southern Xinjiang of Xinjiang Corps, Alar, China; 3 State Key Laboratory for Crop Stress Resistance and High-Efficiency Production, College of Agronomy, Northwest A&F University, Yangling, Shaanxi, China; 4 Institute of Water Saving Agriculture in Arid Regions of China, Northwest A&F University, Yangling, Shaanxi, China

**Keywords:** advanced wheat lines, arid region, joint validation, membership function, PCA, TOPSIS, yield, inter-annual consistency

## Abstract

In arid-region wheat breeding, environmental fluctuations are large, and screening based on single traits or single-year data is often inaccurate. To address these challenges, this study constructed a multi-model collaborative evaluation system to accurately screen advanced lines for high yield potential and inter-annual consistency. Based on 10 yield traits evaluated in 52 advanced wheat lines over two growing seasons, we utilized systematic clustering to classify lines and stepwise regression to pinpoint core contributing traits. Subsequently, principal component analysis (PCA), membership functions, and Technique for Order Preference by Similarity to Ideal Solution (TOPSIS) were integrated for joint validation, enabling the robust identification of lines with superior and inter-annually consistent yield potential. The results showed that: (1) TD-48 and TD-26 ranked among the top ten across all three evaluation methods in both experimental years, showing strong potential for high yield and inter-annual consistency. (2) Cluster analysis divided all tested lines into six categories: High-yield and inter-annually consistent type, High-yield but inter-annually fluctuating type, High-yield type, Medium-yield type, Low-yield type, and Low-yield sensitive type. TD-48 and TD-26 were classified into the high-yield and inter-annually consistent group. (3) A predictive regression model was established via stepwise regression: D = 0.16 + 0.294GY+0.123GWS+0.064TKW+0.107GNP+0.054SNP+0.018BSSN (R^2^=0.983, P<0.001). Accordingly, grain yield (GY in t/ha), grain weight per spike (GWS in g), thousand kernel weight (TKW in g), grain number per plant (GNP), spike number per plant (SNP), and basal sterile spikelet number (BSSN) were identified as the core evaluation indicators. The multi-model consensus screening system established in this study can effectively overcome the limitations of a single-method evaluation. Providing reliable methodological support and valuable candidate germplasm resources for the accurate evaluation and efficient screening of breeding materials in arid regions.

## 1 Introduction

Wheat (Triticum aestivum L.) is one of the most important food crops globally, feeding approximately 35% of the world’s population (IDRC, 2010), and is a key source of energy, starch, and dietary fiber for humans (Shewry and Hey, 2015; P and Joye, 2020). Although global wheat production reached 798.9 million tons in 2023, covering a harvested area of 220.4 million hectares (Kumar et al., 2026; FAO, 2023), under the dual pressures of climate change (Hickey et al., 2019) and sustained population growth, global wheat production is projected to increase by approximately 60% from current levels by 2050 to meet the demand of 9 billion people (Tester and Langridge, 2010; Fischer et al., 2014). Therefore, continuously improving wheat productivity is the fundamental way to ensure global food security. However, the current average genetic gain rate is only 0.9%, far below the required 2.4% (Ray et al., 2013). This increasingly widening yield gap makes it particularly urgent to accelerate genetic progress through breeding technology innovation.

In the breeding process, advanced breeding lines are the key link connecting genetic resources and commercial cultivars, with the highest selection efficiency and cost-effectiveness (Brennan and Martin, 2007; Studnicki et al., 2019). Especially in the early generations of selection, they can significantly increase genetic progress (Bonnett et al., 2021). Yield potential refers to the level of a single agronomic trait, namely grain yield (GY, t/ha), representing the intrinsic productive capacity under specific conditions (Murchie et al., 2023; Andrade et al., 2026). However, the full expression of Advanced Breeding Lines yield potential is strongly constrained by genotype-environment interaction (G×E) (Allard and Bradshaw, 1964). In regions with significant environmental fluctuations such as arid and semi-arid regions, this severely hinders the precise identification and stable utilization of exlit lines. Quantifying the genetic basis of these traits is therefore paramount for effective selection. Broad-sense heritability (*H^2^*), estimated on an entry-mean basis across years using variance components from the random-effects model fitted to the combined dataset, serves as a critical indicator for breeders to gauge the reliability of phenotypic selection (Schmidt et al., 2019). Traits with high *H^2^*, such as TKW, allow for more consistent selection gains across environments compared to those with low *H^2^*, where environmental noise often masks genetic potential (Henkrar et al., 2025). Despite its fundamental importance in predicting breeding outcomes, explicit estimates of variance components and heritability are frequently absent from multi-trait evaluation studies, limiting the interpretation of genotype-by-environment interactions. In this study, “comprehensive performance” was employed as a multivariate metric that integrates yield traits across different years through the combined application of PCA, membership function, and TOPSIS, thereby enabling a more comprehensive evaluation of both productivity and inter-annual consistency. Herein, “overall performance” and “comprehensive yield potential” are used interchangeably with “comprehensive performance” (Tian et al., 2024; Wang et al., 2025; Shi et al., 2026) Therefore, establishing an efficient screening system that can objectively evaluate the comprehensive performance of tested lines while considering inter-annual consistency is crucial for shortening the breeding cycle and releasing the yield potential.

However, there are generally two major bottlenecks in the existing advanced breeding lines evaluation methods, which limit their selection efficiency and reliability. Firstly, traditional strategies relying on single traits or visual screening often ignore the combined effect of multiple traits on yield, potentially leading to the omission of genotypes with the best overall adaptability. Although multi-criteria decision-making methods have been introduced to integrate multi-dimensional phenotypic information (Chen et al., 2012a; Maji, 2012; Hadikurniawati et al., 2019), most studies only apply a single model, such as PCA (Hu et al., 2024), lacking joint validation and consensus analysis among different methods, which raises doubts about the robustness of the evaluation results. Secondly, existing evaluations are mostly based on data from a single year or a single environment, generally ignoring the impact of interannual environmental fluctuations on the inter-annual consistency of variety performance, which is a core attribute for the successful promotion of cultivars in arid regions. These dual deficiencies in methodological rigor and inter-annual consistency assessment lead to a disconnection between advanced evaluation tools and the efficient selection requirements in breeding practices.

To address these challenges, this study has developed a comprehensive evaluation framework for candidate lines that integrates multi-model joint validation and inter-annual consistency evaluation, aiming to achieve precise selection of breeding materials. Concretely, to more reliably identify high-yielding and inter-annually consistent lines and mitigate potential biases arising from single-model or single-environment assessments, we constructed a multi-algorithm consensus evaluation system by integrating PCA, membership function method, and TOPSIS (Fei et al., 2025), while systematically incorporating inter-annual consistency analysis to account for environmental fluctuations across growing seasons. PCA can be used to evaluate the weights of each indicator and extract the main components that affect all variables (Tahjib-Ul-Arif et al., 2018; Ahmad et al., 2020), and combining the membership function, TOPSIS model, and related analysis methods can more accurately and reliably evaluate the performance of plants in different environments (Sun et al., 2021). Specifically, all three methods were based on the same preprocessing step: the min-max normalization was applied to convert all trait values into dimensionless indices in the [0,1] range, with negative indicators being positively transformed beforehand. Based on this standardized matrix, three parallel evaluation approaches were employed: (1) PCA was used to extract core principal components through orthogonal transformation, and a comprehensive score was calculated by weighting the component scores by their variance contribution rates, effectively overcoming the limitations of single-index evaluations that neglect trait associations and overall genotype performance (Alshaharni et al., 2025); (2) the coefficient of variation method was applied to assign weights to each trait, and a weighted sum score (D value) was computed to rank candidate lines (Chen et al., 2012b; Gholizadeh et al., 2022); (3) the entropy-weighted TOPSIS method was adopted, in which entropy weights were assigned to traits, and the relative closeness to the ideal solution was calculated to achieve a comprehensive multi-trait ranking (Gholizadeh Vazvani et al., 2025).The synergistic use and cross-validation of these three methods enable a more comprehensive and reliable evaluation of line performance across years. Detailed mathematical formulations are provided in Section 2.3. Based on the data of 10 key agronomic traits of 52 advanced wheat lines obtained from two consecutive years of field trials in a typical arid ecological area in southern Xinjiang, combined with stepwise regression analysis, this study aimed to: (i) Establish a robust multi-dimensional comprehensive evaluation of the lines identification system for high-yield and inter-annually consistent wheat lines based on the PCA, membership function, and TOPSIS. (ii) Based on the optimal comprehensive evaluation value (D value), a stepwise regression prediction model for key agronomic traits was constructed to quantify the contribution of each trait to the overall yield potential and elucidated the core trait composition determining high-yielding and inter-annually consistent wheat. (iii) Integrating the evaluation results of the three methods for joint validation and screening high-yield and inter-annually consistent lines with consistent performance across multiple trials that are suitable for cultivation in arid regions.

## 2 Materials and methods

### 2.1 Description of experimental materials and site

The experiment was conducted in the field during the 2023–2024 and 2024–2025 growing seasons at the South Xinjiang Industry-Academia Integration Modern Agriculture Training Base of Tarim University. 52 advanced wheat lines (**Table 1**) were used as the experimental lines, with Xindong 60 (the control variety of the South Xinjiang regional trial) serving as the control. The field experiment adopted a randomized block design for comparing the plots. Each plot was 9.3 meters long and 1.8 meters wide, with a row spacing of 20 cm. Each line was replicated three times. Each plot was managed in accordance with the conventional winter wheat cultivation practices in South Xinjiang, ensuring that all environmental factors were consistent while maintaining the same cultivation measures to allow the genetic potential of the lines to be fairly demonstrated.

**Table 1:** List of wheat lines used for testing.

Number	Line	Number	Line	Number	Line	Number	Line
1	TD-1	14	TD-14	27	TD-26	40	TD-39
2	TD-2	15	TD-15	28	TD-27	41	TD-40
3	TD-3	16	TD-16	29	TD-28	42	TD-41
4	TD-4	17	TD-17	30	TD-29	43	TD-42
5	TD-5	18	TD-18 (R)	31	TD-30	44	TD-43
6	TD-6	19	TD-18 (W)	32	TD-31	45	TD-44
7	TD-7	20	TD-19	33	TD-32	46	TD-45
8	TD-8	21	TD-20	34	TD-33	47	TD-46
9	TD-9	22	TD-21	35	TD-34	48	TD-48
10	TD-10	23	TD-22	36	TD-35	49	TD-51
11	TD-11	24	TD-23	37	TD-36	50	TD-52
12	TD-12	25	TD-24	38	TD-37	51	TD-54
13	TD-13	26	TD-25	39	TD-38	52	Xindong 60

“TD-” followed by a numerical code denotes the advanced breeding lines developed by our laboratory via continuous selection over multiple years (where TD stands for Tarim University). The numbers 1 through 52 correspond to the identifiers of all 52 advanced wheat lines.

A total of 52 experimental entries were evaluated, and the results are presented in **Table 1**.

### 2.2 Determination of traits and methods

Five representative plants were randomly selected and harvested from the central rows of each plot to measure individual plant traits. Relevant morphological traits were measured as follows: spike length (SL in cm; measured from the base of the spike to the tip, excluding awns if any), spike number per plant (SNP), spikelet number per spike (SNS), basal sterile spikelet number (BSSN), grain number per spike (GNS), grain weight per spike (GWS in g), grain weight per plant (GWP), thousand kernel weight (TKW; was measured by threshing each spike and counting the number of kernels), grain number per plant (GNP), and Means of grain yield (GY in t/ha).

### 2.3 Statistical analysis

#### 2.3.1 Estimates of broad-sense heritability (H^2^) for key agronomic traits

Broad-sense heritability (H^2^) was estimated on an entry-mean basis across years using variance components from the random-effects model fitted to the combined dataset.

Variance components were derived from mean squares as follows:

(1) $$\sigma_{g}^{2}=(MS_{G}-MS_{G\times Y})/(n_{Y}\cdot r)$$

(2) $$\sigma_{g\times y}^{2}=(MS_{G\times Y}-MS_{E})/r$$

(3) $$\sigma_{e}^{2}=MS_{E}$$

In Equations (1–3), MSG is the genotype mean square, MSG×Y is the genotype×year mean square, MSE is the residual mean square, nY is the number of years, and r is the number of replications per year. Following Equation (4), the across-year *H^2^* was then computed as:

(4) $$H^{2}=\frac{\sigma_{g}^{2}}{\sigma_{g}^{2}+\sigma_{g\times y}^{2}/n_{Y}+\sigma_{e}^{2}/(n_{Y}\cdot r)}$$

Following preliminary correlation analysis, an integrated evaluation framework comprising PCA, the membership function method, and TOPSIS was constructed to quantify the comprehensive performance of wheat lines. The specific calculation steps are outlined below. All data represent were compiled using Microsoft Excel 2021 (Microsoft Corp., Redmond, WA, USA).

#### 2.3.2 Correlation analysis

Correlation analysis was conducted using the Correlation Plot plugin in Origin 2024 (OriginLab Corp., Northampton, MA, USA). Pearson’s correlation coefficients were calculated, with significance levels set at α = 0.05 and α = 0.01 (two-tailed test). The results were visualized as a heatmap showing both correlation coefficients and significance levels (Zheng et al., 2023).

#### 2.3.3 Principal component comprehensive score

(5) $${\text{Z}}_{\text{ij}}=\frac{{\text{X}}_{\text{ij}}-{\text{X}}_{\text{j}\min}}{{\text{X}}_{\text{j}\max}-{\text{X}}_{\text{j}\min}}$$

(6) $${\text{PC}}_{\text{m}}={\text{w}}_{\text{m}1}{\text{Z}}_{1}+{\text{w}}_{\text{m}2}{\text{Z}}_{2}+\cdots +{\text{w}}_{\text{mp}}{\text{Z}}_{\text{p}}$$

(7) $${\text{PC}}_{\text{mi}}={\text{w}}_{\text{m}1}{\text{z}}_{\text{i}1}+{\text{w}}_{\text{m}2}{\text{z}}_{\text{i}2}+\cdots +{\text{w}}_{\text{mp}}{\text{z}}_{\text{ip}},\text{ m}=1,\ldots ,\text{k}$$

(8) $${\text{F}}_{\text{i}}=\frac{\alpha_{1}}{{\text{C}}_{\text{k}}}\cdot {\text{PC}}_{1\text{i}}+\frac{\alpha_{2}}{{\text{C}}_{\text{k}}}\cdot {\text{PC}}_{2\text{i}}+\cdots +\frac{\alpha_{k}}{{\text{C}}_{\text{k}}}\cdot {\text{PC}}_{\text{ki}}$$

Data preprocessing and principal component analysis were conducted as follows. Firstly, use Equation 5 to perform data standardization processing on each indicator. In this study, all subsequent comprehensive evaluation methods—such as the membership function, TOPSIS, stepwise regression—are preceded by standardization using this method. Secondly, use Equation 6 to calculate the m principal component. Then, use Equation 7 to calculate the score of the i sample on the m principal component. Finally, use Equation 8 to calculate the comprehensive score Fi of each sample. X_ij_ represents the original observation value of the i sample on j variable, X_j min_ and X_j max_ represent the minimum and maximum values of the j variable respectively, w_m1_, w_m2_, …, w_mp_ are the feature vectors corresponding to the m principal component, α_1_, α_2_, …, α_k_ are the eigenvalues of each principal component, and C_k_ is the sum of the eigenvalues of the first k principal components.

#### 2.3.4 Fuzzy function comprehensive evaluation

(9) $$\mu_{\text{ij}}=\frac{{\text{X}}_{\text{ij}}-{\text{X}}_{\text{j}\min}}{{\text{X}}_{\text{j}\max}-{\text{X}}_{\text{j}\min}}$$

(10) $${\text{W}}_{\text{j}}=\frac{{\text{V}}_{\text{j}}}{\sum_{\text{j}=1}^{\text{J}}{\text{V}}_{\text{j}}}$$

(11) $$\text{D}=\sum_{\text{i}}^{\text{j}}\left(\mu_{\text{ij}}{\text{W}}_{\text{j}}\right)$$

The data of each indicator is standardized using Equation 9, the weights are processed using Equation 10, and the comprehensive evaluation D value of the output is calculated using Equation 11. Xij represents the measured value of the i sample in the j indicator; Xjmax and Xjmin respectively represent the maximum and minimum values of the j indicator; where Wi denotes the weight of the i-th index among all indices, and Pi represents the contribution rate of the i-th comprehensive index.

#### 2.3.5 TOPSIS Model

The data is standardized using Equations 12, 13; the objective weights of each indicator are calculated using Equations 14, 15 through the entropy weight method; the positive and negative ideal solutions of the evaluation system are determined using Equations 16, 17; then, the weighted Euclidean distances between each evaluated object and the positive and negative ideal solutions are calculated using Equations 18, 19; finally, the relative closeness degree Ci between each evaluated object and the ideal solution is calculated using Equation 20. x_ij_ represents the measured value of the i sample in the j indicator; max(x_ij_) and min(x_ij_) represent the maximum and minimum values of the j indicator respectively; m is the total number of evaluation objects; the information entropy ej measures the dispersion degree of the value of the j indicator; W_j_ is the weight; X^+^ and X^−^ represent the positive indicator set and the negative indicator set respectively; ${\text{S}}_{\text{i}}^{+}$ and ${\text{S}}_{\text{i}}^{-}$ represent the distance between the i object and the optimal solution and the worst solution respectively; the relative closeness degree Ci comprehensively reflects the relative position of the evaluated object to the positive and negative ideal solutions. The larger the Ci value, the closer the object is to the positive ideal solution and the farther it is from the negative ideal solution, and its overall performance is better.

(12) $${\text{x}}^{\prime }=\frac{{\text{x}}_{\text{ij}}-\min({\text{x}}_{\text{ij}})}{\max({\text{x}}_{\text{ij}})-\min({\text{x}}_{\text{ij}})}$$

(13) $${\text{x}}^{\prime }=\frac{\max({\text{x}}_{\text{ij}})-{\text{x}}_{\text{ij}}}{\max({\text{x}}_{\text{ij}})-\min({\text{x}}_{\text{ij}})}$$

(14) $${\text{p}}_{\text{ij}}=\frac{{\text{x}}_{\text{ij}}^{\prime }}{\sum_{\text{i}=1}^{\text{m}}{\text{x}}_{\text{ij}}^{\prime }},\;{\text{e}}_{\text{j}}=-\frac{1}{\ln(\text{m})}\sum_{\text{i}=1}^{\text{m}}{\text{p}}_{\text{ij}}\ln({\text{p}}_{\text{ij}})$$

(15) $${\text{w}}_{\text{j}}=\frac{\left(1-{\text{e}}_{\text{j}}\right)}{\sum_{\text{j}=1}^{\text{n}}\left(1-{\text{e}}_{\text{j}}\right)}$$

(16) $${\text{X}}^{+}=(\max{\text{x}}_{\text{i}1},\max{\text{x}}_{\text{i}2},\ldots ,\max{\text{x}}_{\text{ij}})$$

(17) $${\text{X}}^{-}=(\min{\text{x}}_{\text{i}1},\min{\text{x}}_{\text{i}2},\ldots ,\min{\text{x}}_{\text{ij}})$$

(18) $${\text{S}}_{\text{i}}^{+}=\sqrt{\sum_{\text{j}=1}^{\text{n}}{\text{w}}_{\text{j}}{\left({\text{x}}_{\text{ij}}-x_{\text{j}}^{+}\right)}^{2}};\text{ i}=1,2,\ldots ;\;0\leq {\text{S}}_{\text{i}}^{+}\leq 1$$

(19) $${\text{S}}_{\text{i}}^{-}=\sqrt{\sum_{\text{j}=1}^{\text{n}}{\text{w}}_{\text{j}}{\left({\text{x}}_{\text{ij}}-x_{\text{j}}^{-}\right)}^{2}};\text{ i}=1,2,\ldots ;\;0\leq {\text{S}}_{\text{i}}^{-}\leq 1$$

(20) $${\text{C}}_{\text{i}}=\frac{{\text{S}}_{\text{i}}^{-}}{{\text{S}}_{\text{i}}^{+}+{\text{S}}_{\text{i}}^{-}}$$

PCA was employed to analyze the yield traits of 52 advanced wheat lines, and the membership function values of each trait were calculated (Zhang et al., 2021). On this basis, the D value for evaluating the comprehensive yield potential of each Line was calculated (Xu et al., 2024; Han et al., 2025a). To establish a rapid evaluation model, the D value of comprehensive yield potential was set as the dependent variable, while 10 yield traits such as spike length and number of spikes per plant were set as independent variables. Through stepwise regression analysis, a parameter index and mathematical equation that have a significant impact on the comprehensive yield of breeding materials were developed (Zhang et al., 2024). These equations and indicators can quickly determine the comprehensive yield level of tested wheat lines. PCA, membership function analysis, and multiple stepwise regression analysis were performed using IBM SPSS Statistics 27 (Armonk, NY, USA). In the PCA, PCs_with eigenvalues >1 were extracted, and varimax rotation was applied to enhance factor loading interpretability (Song et al., 2023). TOPSIS analysis was performed using Excel 2021. Graphs were drawn using Origin 2024 software (Origin 2024).

## 3 Results

### 3.1 Descriptive statistics of agronomic traits in 52 advanced wheat lines

**Figure 1:**
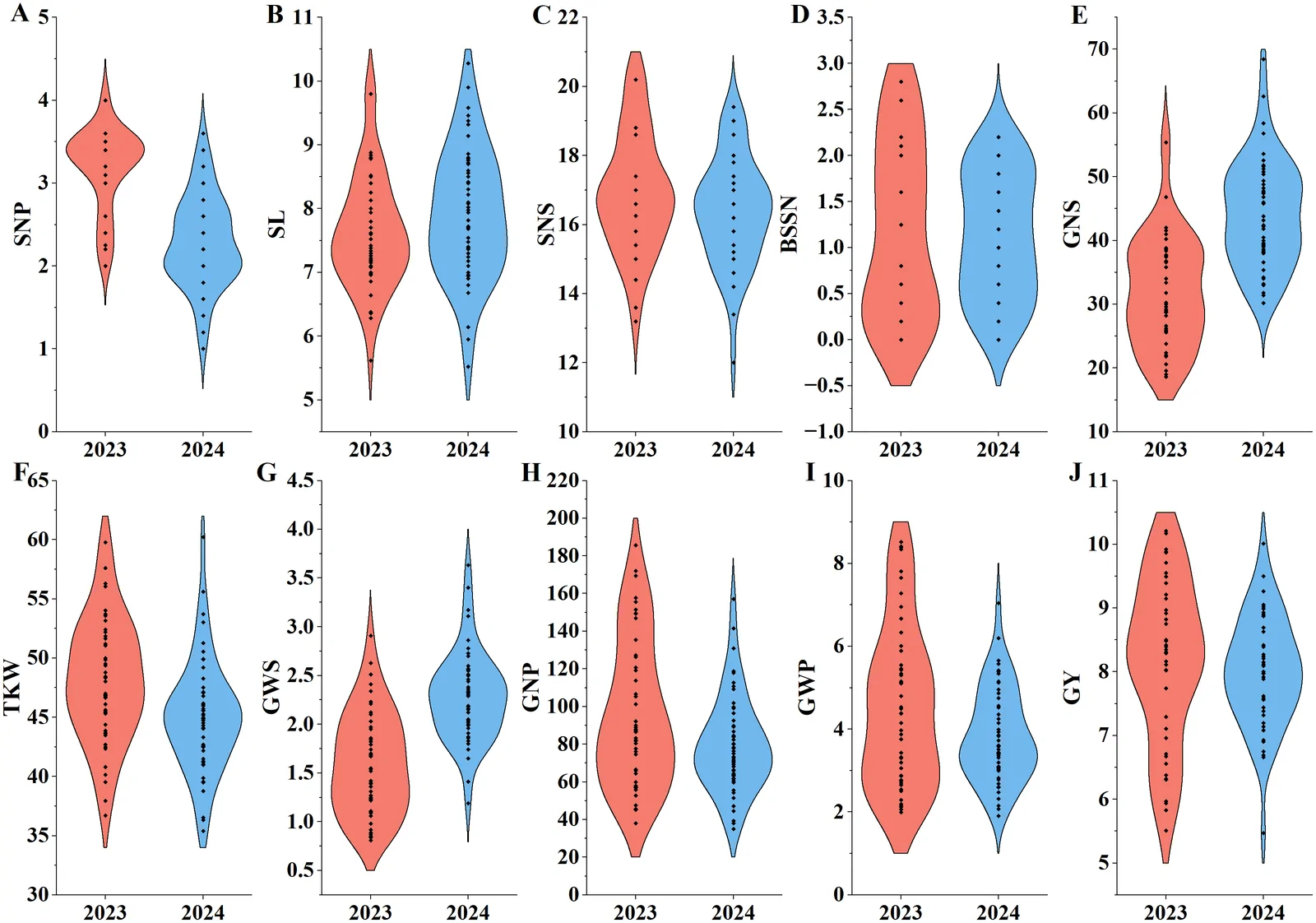
Descriptive statistics of 52 tested wheat lines in 2023 and 2024. **(A–J)** Performance of agronomic traits across two experimental years. “TD-” followed by a numerical code denotes the advanced breeding lines developed by our laboratory via continuous selection over multiple years (where TD stands for Tarim University). **(A)** SNP, spike number per plant; **(B)** SL, spike length (cm); **(C)** SNS, spikelet number per spike; **(D)** BSSN, basal sterile spikelet number; **(E)** GNS, grain number per spike; **(F)** TKW, thousand kernel weight (g); **(G)** GWS, grain weight per spike (g); **(H)** GNP, grain number per plant; **(I)** GWP, grain weight per plant (g); **(J)** GY, grain yield (t/ha).

This study conducted phenotypic identification and descriptive statistical analysis on 10 key yield-related traits of 52 test materials over two consecutive growing seasons (2023 and 2024). The results are shown in **Figure 1**; **Supplementary Table 2**. Extensive genetic variation was observed for each traits within the population during both years. In 2023, the coefficients of variation (CVs) ranged from 10.13% to 81.57%, with a mean of 28.49% (**Supplementary Table 2**); In 2024, the CVs varied between 9.42% and 61.40%, averaging 22.85% (**Supplementary Table 2**). Among them, the BSSN exhibited the greatest variability, with CVs reaching 81.57% and 61.40% in 2023 and 2024 respectively (**Supplementary Table 2**), and the elevated CV in 2023 (81.57%) was primarily driven by greater dispersion and a cluster of near-zero values, as further illustrated by the boxplot and histogram in **Supplementary Figures 1A, 1B**, indicating significant differences in floret-set stability among different lines which is a key trait for screening high-yield and inter-annually consistent lines. Furthermore, a sensitivity analysis confirmed that this high variation originates from the intrinsically left-skewed distribution of BSSN rather than from outliers or measurement errors, thereby confirming the reliability of the BSSN dataset for subsequent genetic analyses (**Supplementary Table 3**). GWP and GNP, together with other important yield-component traits including GNS and GWS, also exhibited high variation, providing sufficient selection potential for high-yield lines. In contrast, traits such as TKW, SNS, and SL exhibited relatively low CVs, indicating that these traits were more consistent within the population. Most traits exhibited reduced CVs in 2024 compared with 2023, which may be related to the influence of inter-annual climate differences on trait expression. Notably, our analysis reveals that (**Supplementary Table 4**): (1)TKW (*H^2^* = 0.957) and SL (*H^2^* = 0.955) exhibited the highest and most consistent genetic signals across years, indicating strong genotype effects. (2) Most agronomic traits—including SNS (0.896), GNP (0.891), GWP (0.872), GNS (0.885), and GY (0.884)—showed high across-year heritability. (3) SNP displayed moderate across-year *H^2^* (0.498), but its within-year estimates varied substantially (0.540 in 2023; 0.824 in 2024), suggesting strong G×E interaction. (4) GY remained highly heritable in both years (0.988 in 2023, 0.978 in 2024), while GWS declined from 0.863 (2023) to 0.632 (2024), further supporting environment-dependent expression (**Supplementary Figure 2**). These results collectively indicate that relying solely on a single trait is insufficient to comprehensively evaluate the overall yield potential of the lines. Therefore, it is necessary to adopt a multi-index comprehensive evaluation method to more scientifically and efficiently identify excellent lines with high-yield potential.

### 3.2 Correlation analysis

**Figure 2:**
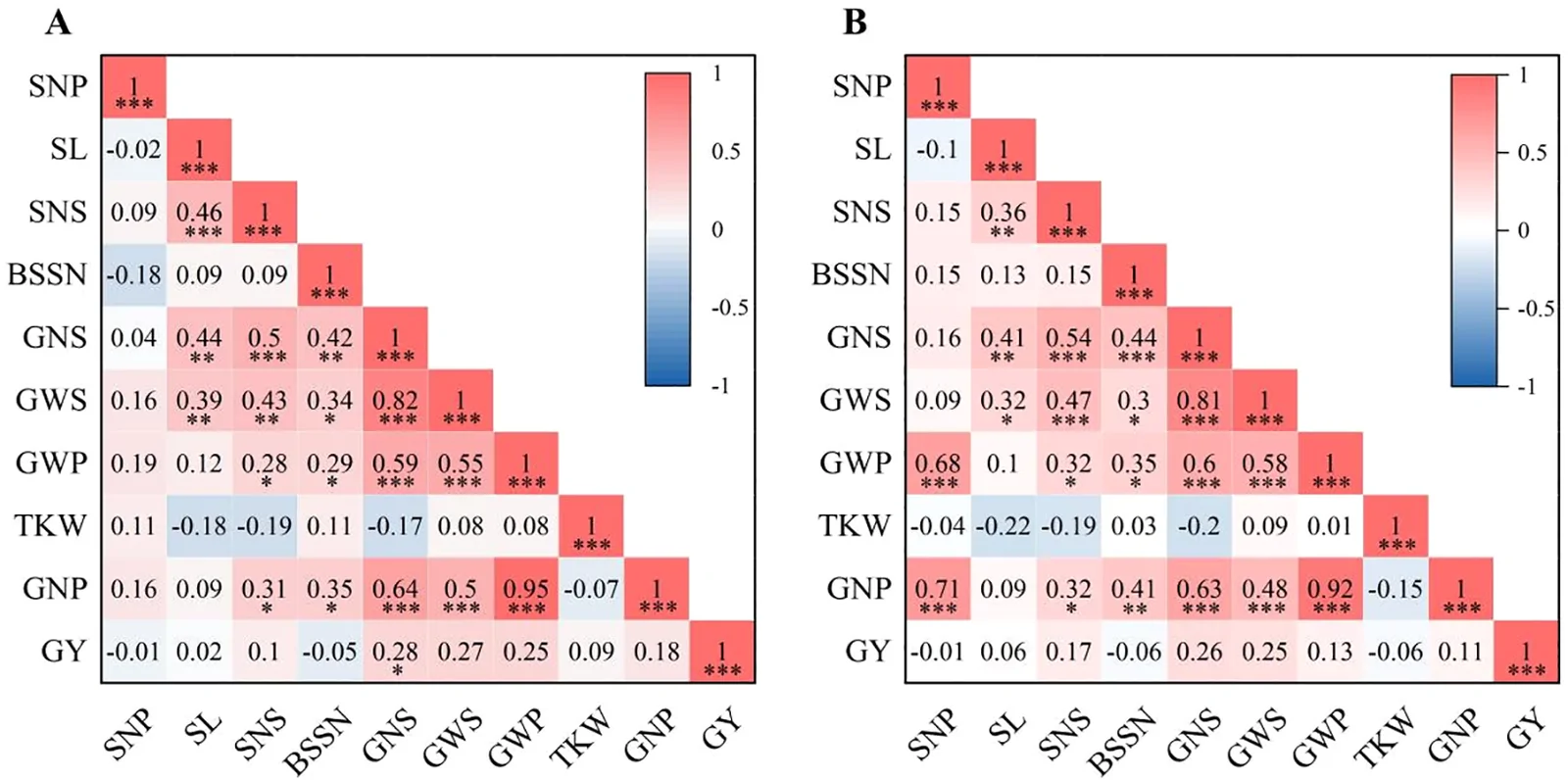
Correlation analysis of the 10 key agronomic traits in 2023 **(A)** and 2024. **(B)**. The larger the circle and the deeper the color, the stronger the correlation. Significant difference: *P<0.05, **P<0.01, ***P<0.001. SNP, spike number per plant; SL, spike length (cm); SNS, spikelet number per spike; BSSN, basal sterile spikelet number; GNS, grain number per spike; TKW, thousand kernel weight (g); GWS, grain weight per spike (g); GNP, grain number per plant; GWP, grain weight per plant (g); GY, grain yield (t/ha).

Based on the statistical analysis results of 10 key yield-related traits in 52 advanced wheat lines, this study further performed correlation analysis on these agronomic traits. The results indicate that there is a widespread correlation network among the yield traits of the experimental entries. In 2023, GWP exhibited a highly significant positive correlation with GNP and GNS. GNS was positively and extremely significantly correlated with GWS and SNS, and GY was positively correlated with key constituent factors including GWP and GWS (**Figure 2A**). In 2024, the positive correlations of GWP with GNP and GNS remained significant; GNS also showed a significant correlation with GWS and SNS, while the correlations between GY and GWP/GWS did not reach statistical significance (**Figure 2B**). Our results suggest that the relationships among yield-related traits are complex and modulated by inter-annual environmental variation. Thus, a single indicator is insufficient to comprehensively evaluate the overall yield potential of the tested lines. Therefore, multivariate methods such as PCA are required to enable a systematic and integrative evaluation.

### 3.3 Statistical validation of comprehensive evaluation indices in 2023 and 2024.

To ensure statistical reliability, the membership function values (D), PCA composite scores, and TOPSIS C-values were initially tested for normality (Shapiro–Wilk) and homoscedasticity (Levene’s test) (**Supplementary Table 5**). The TOPSIS C-values violated both assumptions in both years (P<0.05), thus necessitating the Kruskal–Wallis H test for varietal effect evaluation. Conversely, the D-values and PCA scores met normality but failed homogeneity of variance (P<0.05), and were therefore analyzed using Welch’s ANOVA. Varietal effects were highly significant across all indices (P<0.001). Specifically, TOPSIS C-values yielded H = 136.913 and 156.460 in 2023 and 2024, respectively (both df=51, P<0.001), while Welch’s F-statistics for D-values and PCA scores ranged from F_51_, 71.946 to F_51_, 72.014 = 6.284 (all P<0.001) (**Supplementary Table 5**). Collectively, these results confirm that all three evaluation systems robustly discriminate among the 52 accessions, justifying subsequent ranking and clustering.

### 3.4 Principal component comprehensive score

A PCA was performed on the agronomic traits of 52 advanced wheat lines over two years. The results are summarized in **Tables 2**–**4**. Four principal components with eigenvalues greater than 1 were extracted in 2023, with a cumulative contribution rate of 65.47%. In 2024, three principal components were extracted, and their cumulative contribution rate reached 65.95%. These components can represent most of the original traits and can be used for subsequent comprehensive evaluation. Prior to PCA, we verified that there were no missing data and confirmed the absence of outliers via boxplot inspection. The suitability of PCA was then assessed using the KMO and Bartlett’s tests. The KMO values were 0.707 (2023) and 0.782 (2024), both exceeding the recommended threshold of 0.6. Bartlett’s test of sphericity was significant for both years (2023:X^2^ = 612.475, 2024:X^2^ = 1208.934, df = 45, p< 0.001), indicating that the variables were sufficiently correlated for PCA (**Supplementary Table 8**).

**Table 2:** The feature vector and contribution rate of each principal component.

Year	Principal component	λ1	λ2	λ3	λ4
2023	Eigenvalue	3.02	1.36	1.10	1.06
	Cumulative contribution rate/%	30.24	43.86	54.90	65.47
	SL	0.21	-0.56	0.07	0.23
	SNP	0.06	0.11	0.64	-0.27
	SNS	0.27	-0.48	0.05	0.14
	BSSN	0.22	0.15	-0.65	0.04
	GNS	0.47	0.00	-0.04	0.05
	GWS	0.41	-0.06	0.03	0.33
	GWP	0.45	0.27	0.07	-0.24
	TKW	-0.03	0.51	0.00	0.57
	GNP	0.45	0.19	-0.03	-0.41
	GY	0.17	0.21	0.39	0.44
2024	Eigenvalue	3.97	1.51	1.11	
	Cumulative contribution rate/%	39.68	54.81	65.95	
	SL	0.26	-0.41	-0.05	
	SNP	0.24	0.59	-0.25	
	SNS	0.32	-0.24	-0.03	
	BSSN	0.28	-0.09	0.26	
	GNS	0.44	-0.21	0.09	
	GWS	0.39	-0.22	0.22	
	GWP	0.42	0.32	-0.01	
	TKW	-0.04	0.22	0.72	
	GNP	0.40	0.34	-0.16	
	GY	0.04	-0.21	-0.52	

SNP, spike number per plant; SL, spike length (cm); SNS, spikelet number per spike; BSSN, basal sterile spikelet number; GNS, grain number per spike; TKW, thousand kernel weight (g); GWS, grain weight per spike (g); GNP, grain number per plant; GWP, grain weight per plant (g); GY, grain yield (t/ha).

**Table 3:** The comprehensive scores of 52 breeding materials in 2023.

Line	Factor 1	Ranking	Factor 2	Ranking	Factor 3	Ranking	Factor 4	Ranking	Score	Ranking
TD-1	-2.06	45	0.92	11.00	-0.66	39	-0.91	43	-0.67	47
TD-2	-0.17	29	0.34	23.00	-0.90	45	-1.42	51	-0.26	32
TD-3	-1.14	37	0.22	27.00	-0.02	25	-0.49	33	-0.37	38
TD-4	-2.19	48	0.84	14.00	0.59	14	0.70	17	-0.41	39
TD-5	2.79	3	-0.22	32.00	-0.38	32	-0.29	27	0.74	6
TD-6	-0.91	35	-0.51	39.00	0.30	20	-0.23	26	-0.34	36
TD-7	-0.62	34	-0.49	38.00	-1.52	52	-0.61	39	-0.49	42
TD-8	-0.12	26	-0.39	34.00	-0.62	38	0.20	22	-0.14	30
TD-9	1.29	13	0.73	15.00	-0.22	29	-0.63	40	0.40	13
TD-10	-1.86	43	-0.05	29.00	-0.94	46	0.88	12	-0.58	45
TD-11	-0.29	32	0.39	22.00	-0.76	44	1.46	4	0.04	24
TD-12	-2.16	47	1.30	4.00	0.20	23	0.87	13	-0.36	37
TD-13	-0.59	33	-0.07	30.00	0.40	17	-0.31	29	-0.18	31
TD-14	0.31	23	-1.40	46.00	-0.46	35	0.30	20	-0.11	29
TD-15	-2.21	49	1.08	6.00	-1.10	47	1.44	5	-0.49	43
TD-16	-1.44	39	1.00	8.00	1.35	3	0.36	18	-0.11	28
TD-17	-0.06	25	0.87	13.00	0.76	10	1.76	1	0.37	15
TD-18 (R)	-1.62	41	-1.80	50.00	0.97	7	-0.52	34	-0.68	48
TD-18 (W)	0.75	19	-0.88	42.00	-0.67	40	0.83	14	0.12	21
TD-19	-2.15	46	-1.25	45.00	0.35	19	-0.90	42	-0.88	51
TD-20	1.19	15	-0.04	28.00	-0.33	31	-0.53	35	0.26	18
TD-21	-3.14	52	-1.13	44.00	-0.67	41	-0.57	38	-1.24	52
TD-22	0.61	21	0.90	12.00	-1.16	48	-1.08	47	0.07	23
TD-23	1.84	8	0.60	18.00	-0.42	34	-0.56	37	0.53	9
TD-24	1.15	16	0.27	26.00	0.69	12	-1.13	48	0.34	17
TD-25	2.02	7	1.73	2.00	-0.48	36	-0.36	31	0.75	5
TD-26	2.15	6	0.99	9.00	1.81	2	0.33	19	1.02	2
TD-27	2.15	5	1.00	7.00	-0.75	43	-0.38	32	0.66	7
TD-28	-0.16	28	-0.84	41.00	-0.20	28	1.64	3	-0.01	26
TD-29	1.06	17	-2.28	51.00	1.31	4	0.81	15	0.24	20
TD-30	-1.77	42	0.65	17.00	-1.23	49	0.94	10	-0.48	41
TD-31	-0.13	27	1.52	3.00	2.10	1	1.00	8	0.51	10
TD-32	1.48	10	-2.91	52.00	-0.70	42	1.05	6	0.08	22
TD-33	-0.21	31	0.51	19.00	-1.41	51	-1.06	45	-0.26	33
TD-34	0.75	18	0.42	21.00	0.52	15	0.06	24	0.35	16
TD-35	1.36	12	-0.28	33.00	-0.18	27	0.75	16	0.43	12
TD-36	0.66	20	-0.40	35.00	1.21	5	0.88	11	0.37	14
TD-37	2.89	2	-1.52	48.00	0.91	9	1.68	2	0.95	4
TD-38	-2.38	50	0.47	20.00	0.66	13	-0.36	30	-0.62	46
TD-39	-1.08	36	-0.47	37.00	0.94	8	-0.30	28	-0.32	35
TD-40	0.23	24	-1.69	49.00	0.21	21	-1.41	50	-0.29	34
TD-41	-0.18	30	0.33	24.00	-0.28	30	0.29	21	-0.01	25
TD-42	-1.56	40	-1.49	47.00	0.20	22	-0.53	36	-0.71	50
TD-43	1.72	9	0.97	10.00	-0.55	37	-0.94	44	0.49	11
TD-44	0.36	22	-0.91	43.00	0.50	16	-0.89	41	-0.05	27
TD-45	1.28	14	0.28	25.00	0.07	24	-1.72	52	0.25	19
TD-46	-1.86	44	-0.71	40.00	0.76	11	-1.06	46	-0.69	49
TD-48	2.46	4	2.01	1.00	0.38	18	0.95	9	1.16	1
TD-51	-2.53	51	1.22	5.00	1.18	6	0.11	23	-0.46	40
TD-52	-1.17	38	-0.09	31.00	-1.28	50	0.05	25	-0.50	44
TD-54	3.91	1	-0.45	36.00	-0.38	33	-1.19	49	0.95	3
Xindong 60	1.37	11	0.70	16.00	-0.09	26	1.03	7	0.61	8

“TD-” followed by a numerical code denotes the advanced breeding lines developed by our laboratory via continuous selection over multiple years (where TD stands for Tarim University).

**Table 4:** The comprehensive scores of 52 breeding materials in 2024.

Line	Factor 1	Ranking	Factor 2	Ranking	Factor 3	Ranking	Score	Ranking
TD-1	-1.61	42	1.04	8	0.74	11	-0.40	40
TD-2	0.28	24	1.20	6	0.18	22	0.31	14
TD-3	-0.32	32	1.83	1	-0.99	44	0.04	24
TD-4	-1.92	47	0.37	19	0.73	12	-0.62	45
TD-5	2.85	2	0.21	23	1.13	5	1.29	1
TD-6	-0.60	35	-0.53	39	-0.44	38	-0.37	38
TD-7	-0.40	33	-0.52	37	0.82	9	-0.15	31
TD-8	-0.30	31	-0.17	31	0.44	14	-0.10	30
TD-9	0.88	15	0.55	16	-0.79	40	0.34	13
TD-10	-1.77	45	-1.66	51	0.18	23	-0.93	49
TD-11	-0.25	29	-1.02	45	0.40	18	-0.21	32
TD-12	-1.82	46	0.89	10	2.31	1	-0.33	36
TD-13	-0.53	34	-0.55	40	-0.88	41	-0.39	39
TD-14	0.53	20	-1.06	46	0.04	26	0.05	23
TD-15	-1.72	43	-0.03	28	2.06	3	-0.46	43
TD-16	-1.38	41	0.57	15	-0.27	35	-0.49	44
TD-17	0.00	26	0.01	27	1.22	4	0.14	19
TD-18(R)	1.02	12	-0.64	41	-0.22	33	0.28	16
TD-18(W)	-0.94	39	-0.10	29	0.40	17	-0.34	37
TD-19	-1.94	48	-0.94	44	-2.04	51	-1.14	51
TD-20	0.84	16	-0.42	35	-0.50	39	0.21	17
TD-21	-2.60	52	-1.12	47	0.33	20	-1.16	52
TD-22	0.90	14	1.05	7	2.14	2	0.76	9
TD-23	1.94	7	0.87	12	-1.22	48	0.76	8
TD-24	-2.33	50	0.12	25	-1.37	50	-1.06	50
TD-25	1.54	8	1.27	5	0.42	15	0.85	5
TD-26	2.06	6	0.54	17	-0.05	29	0.89	4
TD-27	2.23	4	1.59	4	-0.93	42	1.02	3
TD-28	-0.11	27	-1.49	50	-0.22	34	-0.29	34
TD-29	0.63	19	-1.28	48	-0.37	36	0.02	26
TD-30	-1.75	44	-0.53	38	0.88	7	-0.68	46
TD-31	-0.26	30	0.72	13	-0.37	37	-0.04	28
TD-32	2.17	5	-3.40	52	-1.25	49	0.21	18
TD-33	0.32	22	1.61	3	0.77	10	0.46	12
TD-34	0.74	17	0.15	24	-2.29	52	0.06	22
TD-35	1.03	11	-0.86	43	0.29	21	0.31	15
TD-36	0.30	23	-0.79	42	-0.10	30	-0.01	27
TD-37	2.26	3	-1.47	49	0.41	16	0.72	10
TD-38	-2.00	49	0.33	21	-0.11	31	-0.76	47
TD-39	-0.64	36	-0.28	33	0.01	28	-0.30	35
TD-40	0.47	21	0.23	22	-1.16	47	0.09	20
TD-41	-0.12	28	0.01	26	0.60	13	0.02	25
TD-42	-0.92	38	-0.40	34	0.17	24	-0.41	41
TD-43	1.49	9	0.90	9	0.36	19	0.77	7
TD-44	0.18	25	-0.15	30	-1.07	45	-0.07	29

“TD-” followed by a numerical code denotes the advanced breeding lines developed by our laboratory via continuous selection over multiple years (where TD stands for Tarim University).

In 2023, the eigenvalue of the first principal component (PC1) was 3.02, with a contribution rate of 30.24%. Thus, it was the primary factor influencing trait variation. PC1 showed high positive loadings for GNS (0.47), GWP (0.45), GNP (0.45) and GWS (0.41). This component integrates the entire developmental process from seedling establishment to biomass accumulation, and effectively characterizes the final grain yield potential of individual wheat plants. Accordingly, PC1 was referred to as the “Single-Plant Productivity Factor” (Factor 1). The second principal component (PC2) had a contribution rate of 13.62%, which was mainly characterized by a high positive loading of thousand kernel weight (TKW, 0.51) and a negative loading of spike length (SL, -0.56). It may reflect the mutual restriction between grain size and spike morphology, and can be interpreted as the “Spike Architecture Factor” (Factor 2). The third principal component (PC3) contributed 11.04%, which was dominated by the positive loading of SNP (0.64) and the negative loading of BSSN (-0.65). It embodied the relationship between population structure and spikelet fertility, and could be regarded as the “Population Structure Factor” (Factor 3). The fourth principal component (PC4) had a contribution rate of 10.57%, with relatively high loadings of grain yield (GY, 0.44) and kernel weight (TKW, 0.57), which could be defined as the “comprehensive yield factor” (Factor 4). The fourth principal component (PC4) had a contribution rate of 10.57%, with relatively high loadings of GY (0.44) and TKW (0.57), which could be defined the “Comprehensive Yield Factor” (Factor 4).

In 2024, a total of 3 principal components were extracted. PC1 had an increased eigenvalue of 3.97, with a contribution rate of 39.68%. Its high-loading traits were highly consistent with the 2023 results, mainly encompassing GNS (0.44), GWP (0.42), GNP (0.40) and GWS (0.39), confirming the core position of single-plant productivity composition and also defined as the “Single-plant Productivity Factor” (Factor 1). PC2 had a contribution rate of 15.13%, with the highest loading observed in SNP (0.59), which independently represented the effective tillering capacity, so it was named the “Tiller and Spike formation factor” (Factor 2). PC3 had a contribution rate of 11.14%, which was highlighted by the high positive loading of TKW (0.72) and the negative loading of GY (-0.52). It reflected the potential restrictive relationship between grain plumpness and yield performance under the environmental condition of this year, and was designated as the “Grain Fullness Factor” (Factor 3). Overall, the PCA results across the two years revealed a highly consistent trait structure. PC1 represented single-plant productivity in both years, which was the most inter-annually consistent genetic structure. The main differences were as follows: in 2023, TKW was distributed in PC2 and PC4, while SNP and BSSN jointly constituted the population structure factor; in 2024, the analysis was simplified to 3 principal components, TKW and GY formed an independent trade-off axis in PC3, and SNP independently constituted PC2. These results indicated that single-plant productivity remained inter-annually consistent across years, while the relationship between grain weight and tillering might vary with annual environmental conditions.

Based on the PCA composite scores, eight lines—TD-5, TD-26, TD-27, TD-37, TD-48, TD-54, TD-23, and TD-25—maintained top-10 rankings across both 2023 and 2024 (**Tables 3**, **4**), demonstrating outstanding comprehensive advantages. In contrast, the check cultivar Xindong 60 (ranked 8th in 2023 but 21st in 2024) and several other lines exhibited marked rank shifts, indicating substantial sensitivity to inter-annual environmental fluctuations. Notably, grain-filling periods (Apri-May) in 2024 and 2025 differed dramatically in precipitation (30.9 mm vs. 0 mm) despite comparable temperatures (**Supplementary Figure 2**). Such stark inter-annual moisture fluctuations likely drove the differential trait responses and structural reorganizations identified via PCA, highlighting the role of drought stress in shaping yield stability.

These results confirm that the PCA-based evaluation system can effectively discriminate genetic potential among lines and identify materials with robust inter-annual stability, providing a reliable reference for subsequent breeding selection.

### 3.5 Membership function

The contribution rate and D value were calculated using PCA (**Table 5**). The membership function values were determined using the membership function method. The weight values of the principal components for 2023 were 46.19%, 20.80%, 16.87%, and 16.14%; for 2024, they were 60.17%, 22.94%, 16.89%, and 13.45%. Using these weights and membership function values, the comprehensive evaluation (D value) of the yield potential of each wheat line was calculated. The membership function values and D values of each line are shown in the **Table 5**. The higher the D value, the stronger the comprehensive yield potential of the line. This method provides an objective approach for evaluating the comprehensive yield potential of wheat lines. The evaluation results show that there are significant differences in yield potential among different lines. TD-26 performed the most outstandingly in both years, maintaining a D-value of 0.7, ranking at the top. TD-48, TD-21, and TD-19 also performed excellently, maintaining consistently high D-values. The D value of the control variety Xindong 60 was 0.62 and 0.77. Conversely, other lines such as TD-21 and TD-19 maintained relatively low D-values, indicating a relatively weak comprehensive yield potential. This method integrates multiple principal component information and combines multi-dimensional yield traits into a single quantitative indicator (D value), providing an effective tool for objectively and systematically evaluating the yield potential of tested wheat lines. The selected high-D value lines can be used as important candidate materials for high-yield breeding.

**Table 5:** Membership function values and D values for yield and agronomic traits of 52 breeding materials across two growing seasons.

	2023									2024						
Line	Comoprehensive inde				Membership function					Cnmoprehensive inde			Membership function			
	X1	X2	X3	X4	μ1	μ2	μ3	μ4	D Value	X1	X2	X3	μ1	μ2	μ3	D Value
TD1	-1.18	0.79	-0.63	-0.88	0.21	0.76	0.29	0.34	0.36	-0.81	0.84	0.70	0.33	0.32	0.67	0.38
TD-2	-0.10	0.29	-0.86	-1.37	0.45	0.65	0.25	0.24	0.42	0.14	0.97	0.17	0.36	0.50	0.69	0.45
TD-3	-0.65	0.19	-0.02	-0.48	0.33	0.63	0.39	0.42	0.42	-0.16	1.48	-0.94	0.54	0.44	0.76	0.56
TD-4	-1.25	0.72	0.56	0.68	0.20	0.74	0.49	0.66	0.44	-0.96	0.30	0.69	0.53	0.29	0.59	0.48
TD-5	1.60	-0.18	-0.36	-0.28	0.81	0.55	0.34	0.46	0.62	1.43	0.17	1.07	0.37	0.74	0.57	0.49
TD-6	-0.52	-0.44	0.28	-0.23	0.36	0.50	0.45	0.47	0.42	-0.30	-0.43	-0.42	0.54	0.41	0.48	0.50
TD-7	-0.35	-0.42	-1.45	-0.59	0.39	0.50	0.14	0.40	0.37	-0.20	-0.42	0.77	0.28	0.43	0.48	0.35
TD-8	-0.07	-0.33	-0.59	0.19	0.45	0.52	0.29	0.56	0.46	-0.15	-0.14	0.42	0.46	0.44	0.52	0.46
TD-9	0.74	0.62	-0.21	-0.61	0.63	0.72	0.36	0.39	0.56	0.44	0.45	-0.75	0.72	0.56	0.61	0.67
TD-10	-1.07	-0.04	-0.90	0.85	0.24	0.58	0.24	0.69	0.38	-0.89	-1.35	0.17	0.72	0.30	0.34	0.56
TD-11	-0.17	0.33	-0.73	1.42	0.43	0.66	0.27	0.81	0.51	-0.13	-0.82	0.38	0.73	0.45	0.42	0.61
TD-12	-1.24	1.11	0.19	0.85	0.20	0.83	0.43	0.69	0.45	-0.91	0.72	2.19	0.46	0.30	0.65	0.46
TD-13	-0.34	-0.06	0.38	-0.30	0.39	0.58	0.46	0.45	0.45	-0.26	-0.45	-0.83	0.67	0.42	0.48	0.58
TD-14	0.18	-1.20	-0.43	0.30	0.51	0.34	0.32	0.58	0.45	0.27	-0.86	0.04	0.68	0.52	0.41	0.60
TD-15	-1.27	0.93	-1.04	1.40	0.20	0.79	0.21	0.80	0.42	-0.86	-0.02	1.95	0.47	0.31	0.54	0.44
TD-16	-0.83	0.85	1.29	0.35	0.29	0.77	0.62	0.59	0.49	-0.69	0.47	-0.26	0.59	0.34	0.61	0.54
TD-17	-0.04	0.74	0.72	1.70	0.46	0.75	0.52	0.87	0.60	0.00	0.01	1.16	0.59	0.47	0.54	0.55
TD-18(R)	-0.93	-1.54	0.93	-0.50	0.27	0.26	0.56	0.41	0.34	0.51	-0.52	-0.21	0.42	0.57	0.46	0.46
TD-18(W)	0.43	-0.76	-0.64	0.80	0.56	0.43	0.29	0.68	0.51	-0.47	-0.09	0.38	0.46	0.38	0.53	0.46
TD-19	-1.24	-1.07	0.34	-0.87	0.20	0.36	0.46	0.34	0.30	-0.97	-0.77	-1.93	0.72	0.28	0.43	0.57
TD-20	0.68	-0.03	-0.31	-0.52	0.61	0.58	0.34	0.41	0.53	0.42	-0.34	-0.48	0.57	0.55	0.49	0.55
TD-21	-1.80	-0.97	-0.64	-0.56	0.08	0.38	0.29	0.40	0.23	-1.30	-0.91	0.32	0.30	0.22	0.41	0.30
TD-22	0.35	0.77	-1.10	-1.04	0.54	0.75	0.20	0.30	0.49	0.45	0.86	2.03	0.04	0.56	0.67	0.27
TD-23	1.06	0.51	-0.40	-0.55	0.69	0.70	0.33	0.40	0.59	0.97	0.70	-1.16	0.74	0.66	0.65	0.71
TD-24	0.66	0.23	0.65	-1.10	0.61	0.64	0.51	0.29	0.55	-1.17	0.09	-1.30	0.67	0.25	0.56	0.55
TD-25	1.16	1.48	-0.45	-0.35	0.72	0.90	0.32	0.44	0.64	0.77	1.03	0.40	0.59	0.62	0.69	0.61
TD-26	1.23	0.85	1.72	0.32	0.73	0.77	0.70	0.58	0.71	1.03	0.44	-0.05	0.75	0.67	0.61	0.71
TD-27	1.23	0.86	-0.72	-0.37	0.73	0.77	0.27	0.44	0.62	1.12	1.29	-0.88	0.60	0.68	0.73	0.64
TD-28	-0.09	-0.72	-0.19	1.59	0.45	0.44	0.36	0.84	0.50	-0.05	-1.21	-0.21	0.75	0.46	0.36	0.62
TD-29	0.61	-1.95	1.24	0.79	0.60	0.18	0.61	0.68	0.53	0.32	-1.04	-0.35	0.56	0.53	0.39	0.53
TD-30	-1.02	0.56	-1.17	0.91	0.25	0.71	0.19	0.70	0.41	-0.88	-0.43	0.83	0.54	0.30	0.48	0.47
TD-31	-0.07	1.30	1.99	0.97	0.45	0.86	0.75	0.71	0.63	-0.13	0.59	-0.35	0.84	0.45	0.63	0.72
TD-32	0.85	-2.49	-0.67	1.02	0.65	0.06	0.28	0.73	0.48	1.09	-2.76	-1.18	0.79	0.68	0.13	0.66
TD-33	-0.12	0.44	-1.33	-1.02	0.44	0.68	0.16	0.31	0.42	0.16	1.31	0.73	0.28	0.50	0.74	0.41
TD-34	0.43	0.36	0.49	0.06	0.56	0.67	0.48	0.53	0.56	0.37	0.12	-2.17	0.84	0.54	0.56	0.73
TD-35	0.78	-0.24	-0.17	0.73	0.63	0.54	0.37	0.66	0.57	0.52	-0.70	0.28	0.62	0.57	0.44	0.58
TD-36	0.38	-0.34	1.15	0.85	0.55	0.52	0.60	0.69	0.57	0.15	-0.64	-0.09	0.59	0.50	0.45	0.55
TD-37	1.66	-1.30	0.86	1.63	0.82	0.31	0.55	0.85	0.68	1.13	-1.19	0.39	0.57	0.69	0.36	0.56
TD-38	-1.36	0.40	0.62	-0.35	0.18	0.67	0.51	0.45	0.38	-1.00	0.27	-0.10	0.52	0.28	0.58	0.48
TD-39	-0.62	-0.41	0.89	-0.29	0.34	0.50	0.55	0.46	0.43	-0.32	-0.22	0.01	0.46	0.41	0.51	0.46
TD-40	0.13	-1.45	0.20	-1.37	0.50	0.28	0.43	0.24	0.40	0.23	0.19	-1.10	0.43	0.52	0.57	0.48
TD-41	-0.10	0.29	-0.27	0.29	0.45	0.65	0.35	0.58	0.49	-0.06	0.01	0.56	0.47	0.46	0.54	0.48
TD-42	-0.90	-1.28	0.19	-0.52	0.28	0.32	0.43	0.41	0.33	-0.46	-0.33	0.16	0.33	0.38	0.49	0.37
TD-43	0.99	0.83	-0.52	-0.92	0.68	0.77	0.31	0.33	0.58	0.75	0.73	0.34	0.46	0.61	0.65	0.53
TD-44	0.21	-0.78	0.48	-0.86	0.51	0.42	0.48	0.34	0.46	0.09	-0.12	-1.01	0.47	0.49	0.52	0.48
TD-45	0.73	0.24	0.06	-1.67	0.63	0.64	0.41	0.18	0.52	0.47	1.31	0.07	0.47	0.56	0.74	0.54
TD-46	-1.07	-0.61	0.72	-1.03	0.24	0.46	0.52	0.31	0.34	-0.59	0.28	0.02	0.39	0.36	0.58	0.41
TD-48	1.41	1.72	0.36	0.92	0.77	0.95	0.46	0.71	0.75	0.73	0.72	0.93	0.62	0.61	0.65	0.62
TD-51	-1.45	1.05	1.12	0.11	0.16	0.81	0.59	0.54	0.43	-1.26	0.56	-0.18	0.63	0.23	0.62	0.54
TD-52	-0.67	-0.07	-1.21	0.05	0.32	0.57	0.19	0.53	0.39	-0.40	-0.16	0.78	0.41	0.39	0.52	0.42
TD-54	2.24	-0.38	-0.36	-1.15	0.95	0.51	0.33	0.28	0.65	1.50	0.37	-0.91	0.54	0.76	0.60	0.60
Xin dong 60	0.79	0.60	-0.09	1.00	0.64	0.72	0.38	0.72	0.62	0.33	-0.38	-1.08	0.94	0.53	0.48	0.77

“TD-” followed by a numerical code denotes the advanced breeding lines developed by our laboratory via continuous selection over multiple years (where TD stands for Tarim University).

**Figure 3:**
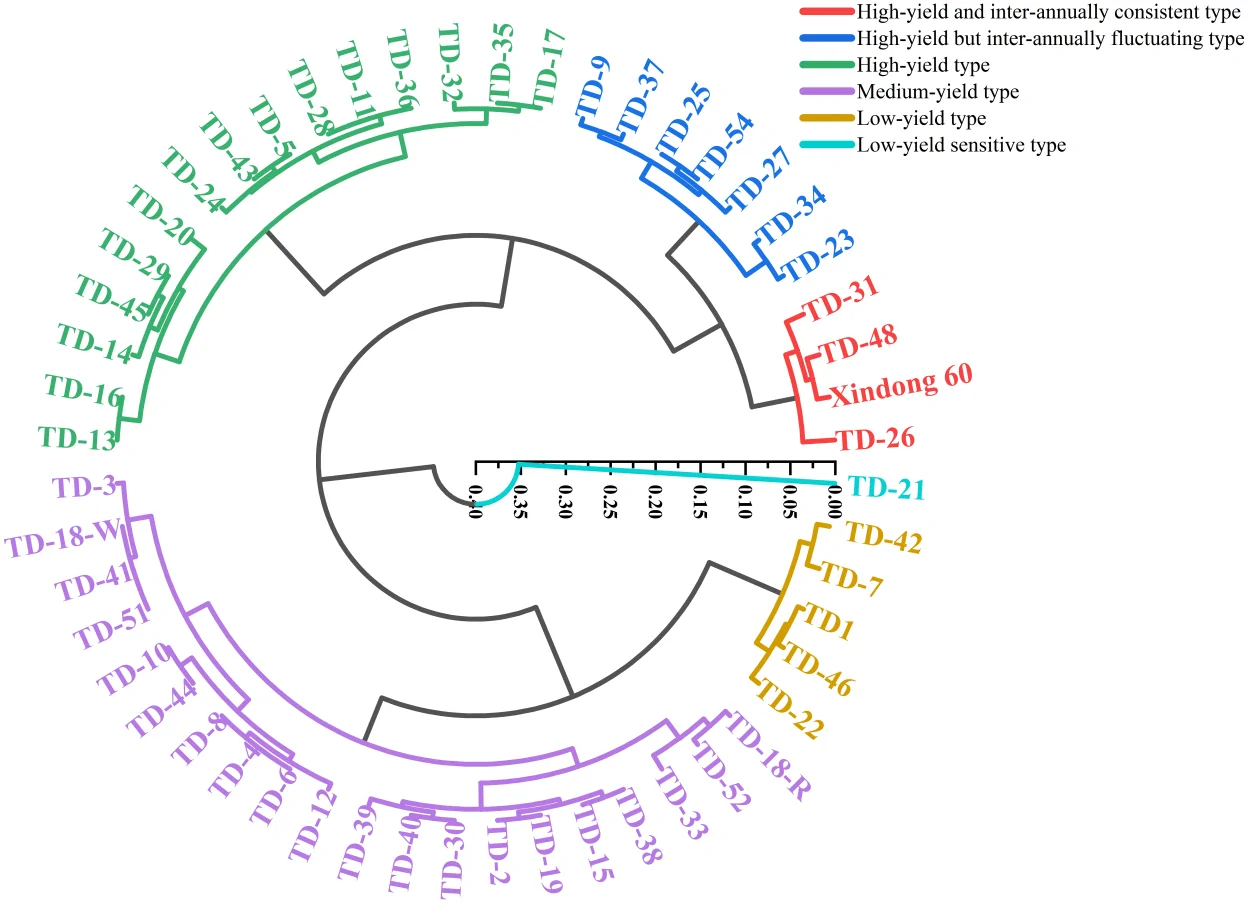
Clustering of 52 advanced wheat lines based on the two-year average D values of the membership function. “TD-” followed by a numerical code denotes the advanced breeding lines developed by our laboratory via continuous selection over multiple years (where TD stands for Tarim University). Different colors represent distinct yield potential groups: red, High-yield and inter-annually consistent type; blue, High-yield but inter-annually fluctuating type; green, High-yield type; purple, Medium-yield type; yellow, Low-yield type; cyan, Low-yield sensitive type.

Based on the two-year mean membership function D values (**Supplementary Table 6**), a systematic cluster analysis was performed on 52 advanced wheat lines using Euclidean distance and the unweighted pair-group method with arithmetic means (UPGMA) (Du et al., 2025). The dendrogram was cut off at a distance threshold of 0.075, resulting in six clusters corresponding to distinct yield potential groups (**Figure 3**). Cluster I (High-yield and inter-annually consistent type) comprised four lines (7.7%), with D-values ranging from 0.68 to 0.71. This group exhibited the most favorable performance across yield-related traits and represents core candidate lines for high-yield and stable-yield breeding programs, possessing high direct utilization value. Cluster II (High-yield but inter-annually fluctuating type) comprised seven lines (13.5%), with D-values ranging from 0.62 to 0.65. While these lines exhibited relatively favorable overall performance, they represent valuable high-yielding germplasm warranting consideration in subsequent breeding programs. Cluster III (High-yield type) included 15 lines (28.8%), with D-values between 0.52 and 0.58. These lines showed relatively superior overall performance and can be prioritized as high-yielding germplasm for further selection and utilization. Cluster IV (Medium-yield type), the largest cluster in this study, contained 20 lines (38.5%) with D-values of 0.40–0.49. Their overall performance was moderate, indicating certain potential for genetic improvement. Cluster V (Low-yield type) comprised five lines (9.6%), with D-values ranging from 0.35 to 0.38. These lines performed poorly overall and were generally characterized by evident yield limitations, resulting in limited direct breeding value. Group VI (Low-Yield Sensitive Type) comprised only TD-21 (1.9%), with a D-value of 0.27. This group showed the poorest overall performance among all clusters, suggesting its practical utility in current breeding programs may be quite limited.

In order to screen and evaluate the comprehensive yield potential of the advanced winter wheat lines, we used the D value as the dependent variable and various agronomic traits as independent variables to develop the best regression equation for predicting the comprehensive yield potential (Zhan et al., 2013). The optimal regression equation was: D = 0.16 + 0.294GY+0.123GWS+0.064TKW+0.107GNP+0.054SNP+0.018BSSN (R^2^=0.983, P<0.001). The results indicated that GY, GWS, TKW, GNP, SNP, and BSSN were the primary determinants of comprehensive performance, collectively explaining 98.3% of the phenotypic variance in the D value. This regression equation provides a robust tool for the preliminary assessment of yield potential during breeding programs. By conducting phenotypic identification only on these six key traits, the tested lines can be preliminarily can be preliminarily classified into High-yield and inter-annually consistent type, High-yield but inter-annually fluctuating type, High-yield type, Medium-yield type, Low-yield type, and Low-yield sen.

**Figure 4:**
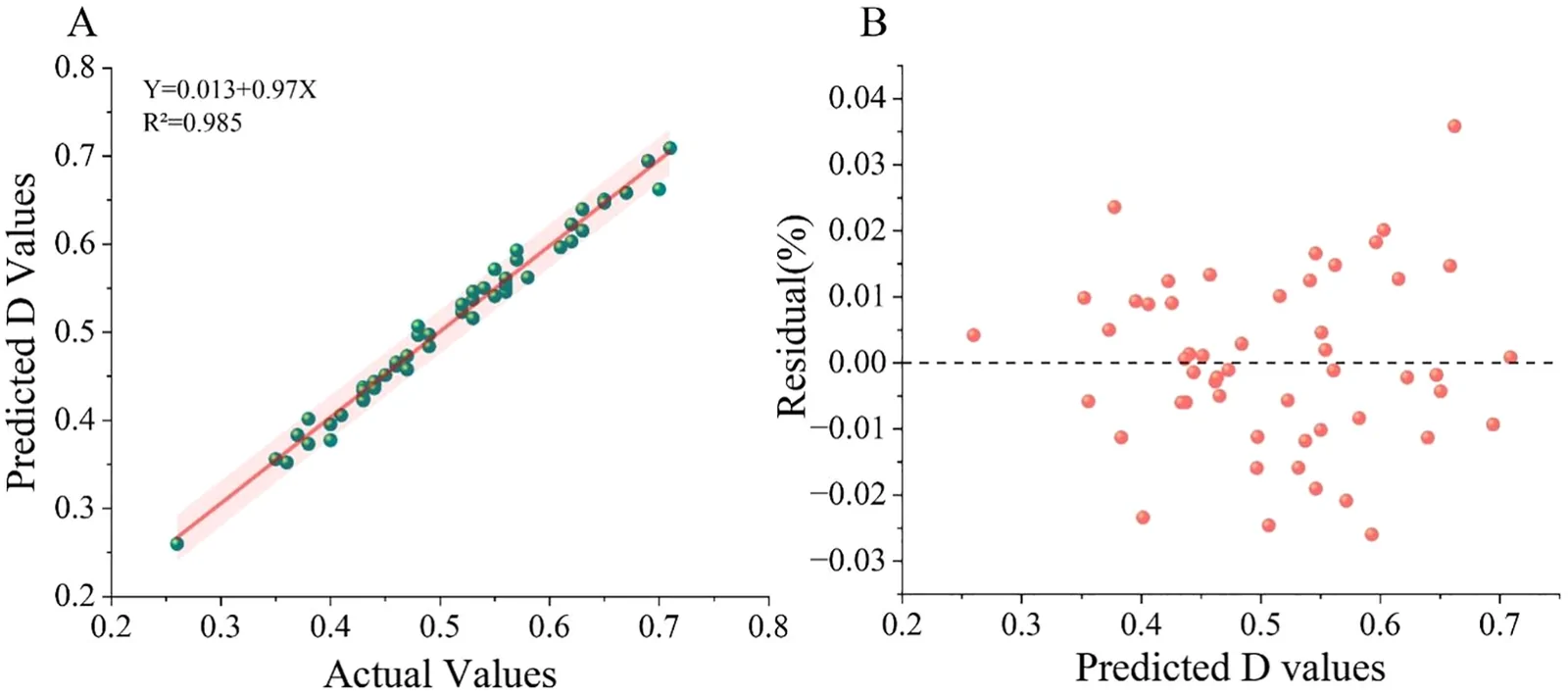
Validation of the stepwise regression model performance. **(A)** Scatter plot of the predicted versus observed D-values, showing high predictive accuracy. **(B)** Residual scatter plot showing the distribution of residuals against predicted D-values, used to assess model fit and variance homogeneity.

Six indicators (GY, GWS, TKW, GNP, SNP, and BSSN) were retained in the final model by the stepwise regression algorithm. Model performance was evaluated through residual analysis and comparison of the predicted versus the observed D-values (**Figure 4**). The close alignment of the predicted and observed values along the diagonal line (R2 = 0.985) indicates high consistency and robustness. The regression results (**Supplementary Table 7**) further confirmed the overall model fit and statistical significance (F = 437.192, ***P < 0.001). These findings suggest that the selected key morphological traits can serve as reliable indicators for the evaluation and screening of high-yielding wheat germplasm based on the comprehensive D-value.

### 3.6 Analysis of yield potential and inter-annual consistency in wheat advanced lines using the TOPSIS Model

The comprehensive evaluation results based on the TOPSIS model (**Table 6**) further verified the differences in the yield potential of each advanced line. This method ranks the lines by calculating the relative closeness degree (C value, 0-1) of each line to the positive and negative ideal solutions. The higher the C value, the better the overall performance. The evaluation results show that there are significant differences in the C values among different lines, and the rankings fluctuate from year to year. TD-54 and TD-27 performed the most outstandingly, ranking first in 2023 and 2024 respectively. TD-54, TD-25, TD-26, and TD-48, among others, remained at the top positions throughout the two years, demonstrating high yield and inter-annual consistency. The control variety Xindong 60 had C values of 0.47 and 0.44 respectively in the two years, ranking within the top 31% and performing well. However, the C value of TD-21 was the lowest in both years, indicating the weakest overall potential; the rankings of TD-10 and TD-19 also remained consistently lower. The TOPSIS method provides another dimension of evaluation results from the perspective of “distance from the ideal solution”. Its ranking trend is basically consistent with the principal component and membership function methods, providing a reliable basis for subsequent selection of high-yield and stable-yield lines.

**Table 6:** The comprehensive assessment results of yield potential for tested wheat lines over two years based on the TOPSIS method.

Line	Euclideandistance (2023)			Euclideandistance (2024)		
	S^+^	S^-^	C	S^+^	S^-^	C
TD-1	0.26	0.16	0.38	0.25	0.14	0.35
TD-2	0.24	0.16	0.40	0.21	0.17	0.45
TD-3	0.30	0.12	0.28	0.21	0.21	0.50
TD-4	0.26	0.13	0.34	0.27	0.11	0.29
TD-5	0.17	0.23	0.58	0.19	0.20	0.51
TD-6	0.24	0.15	0.38	0.25	0.13	0.34
TD-7	0.29	0.13	0.30	0.26	0.13	0.33
TD-8	0.29	0.12	0.29	0.25	0.12	0.33
TD-9	0.23	0.18	0.44	0.21	0.17	0.45
TD-10	0.33	0.09	0.22	0.31	0.10	0.24
TD-11	0.29	0.14	0.32	0.26	0.14	0.35
TD-12	0.26	0.15	0.36	0.27	0.11	0.30
TD-13	0.24	0.16	0.39	0.25	0.13	0.35
TD-14	0.24	0.17	0.41	0.24	0.15	0.39
TD-15	0.30	0.13	0.30	0.28	0.12	0.30
TD-16	0.22	0.18	0.44	0.25	0.14	0.36
TD-17	0.24	0.18	0.43	0.24	0.13	0.35
TD-18(R)	0.26	0.14	0.35	0.22	0.17	0.43
TD-18(W)	0.25	0.16	0.40	0.25	0.13	0.34
TD-19	0.27	0.13	0.33	0.27	0.11	0.30
TD-20	0.22	0.18	0.45	0.23	0.15	0.40
TD-21	0.35	0.07	0.16	0.31	0.08	0.21
TD-22	0.21	0.19	0.48	0.22	0.16	0.42
TD-23	0.24	0.19	0.45	0.19	0.19	0.49
TD-24	0.24	0.17	0.42	0.27	0.11	0.29
TD-25	0.23	0.21	0.49	0.19	0.20	0.50
TD-26	0.20	0.22	0.53	0.20	0.19	0.49
TD-27	0.28	0.19	0.41	0.17	0.23	0.57
TD-28	0.30	0.13	0.30	0.27	0.13	0.33
TD-29	0.23	0.16	0.41	0.24	0.13	0.35
TD-30	0.32	0.11	0.26	0.29	0.11	0.26
TD-31	0.20	0.20	0.50	0.23	0.16	0.41
TD-32	0.30	0.15	0.33	0.26	0.16	0.38
TD-33	0.24	0.16	0.40	0.21	0.19	0.47
TD-34	0.22	0.17	0.44	0.21	0.19	0.47
TD-35	0.22	0.18	0.45	0.23	0.15	0.39
TD-36	0.23	0.17	0.43	0.24	0.14	0.36
TD-37	0.22	0.20	0.48	0.22	0.16	0.42
TD-38	0.26	0.13	0.33	0.27	0.11	0.28
TD-39	0.25	0.14	0.37	0.25	0.12	0.33
TD-40	0.24	0.17	0.42	0.21	0.18	0.46
TD-41	0.24	0.16	0.40	0.24	0.14	0.36
TD-42	0.26	0.13	0.33	0.26	0.11	0.30
TD-43	0.21	0.20	0.49	0.20	0.18	0.47
TD-44	0.22	0.16	0.42	0.23	0.14	0.38
TD-45	0.22	0.19	0.46	0.20	0.20	0.50
TD-46	0.26	0.15	0.35	0.25	0.12	0.32
TD-48	0.21	0.21	0.50	0.20	0.18	0.48
TD-51	0.27	0.13	0.33	0.27	0.11	0.28
TD-52	0.29	0.12	0.28	0.26	0.12	0.31
TD-54	0.21	0.23	0.54	0.17	0.22	0.56
Xindong 60	0.22	0.19	0.47	0.22	0.17	0.44

S^+^, positive ideal solution; S^-^, Distance to negative ideal; C, Composite scoring index;”TD-” followed by a numerical code denotes the advanced breeding lines developed by our laboratory via continuous selection over multiple years (where TD stands for Tarim University).

### 3.7 Integrating PCA, membership functions, and TOPSIS for joint validation of core elite lines via Venn diagram analysis

**Figure 5:**
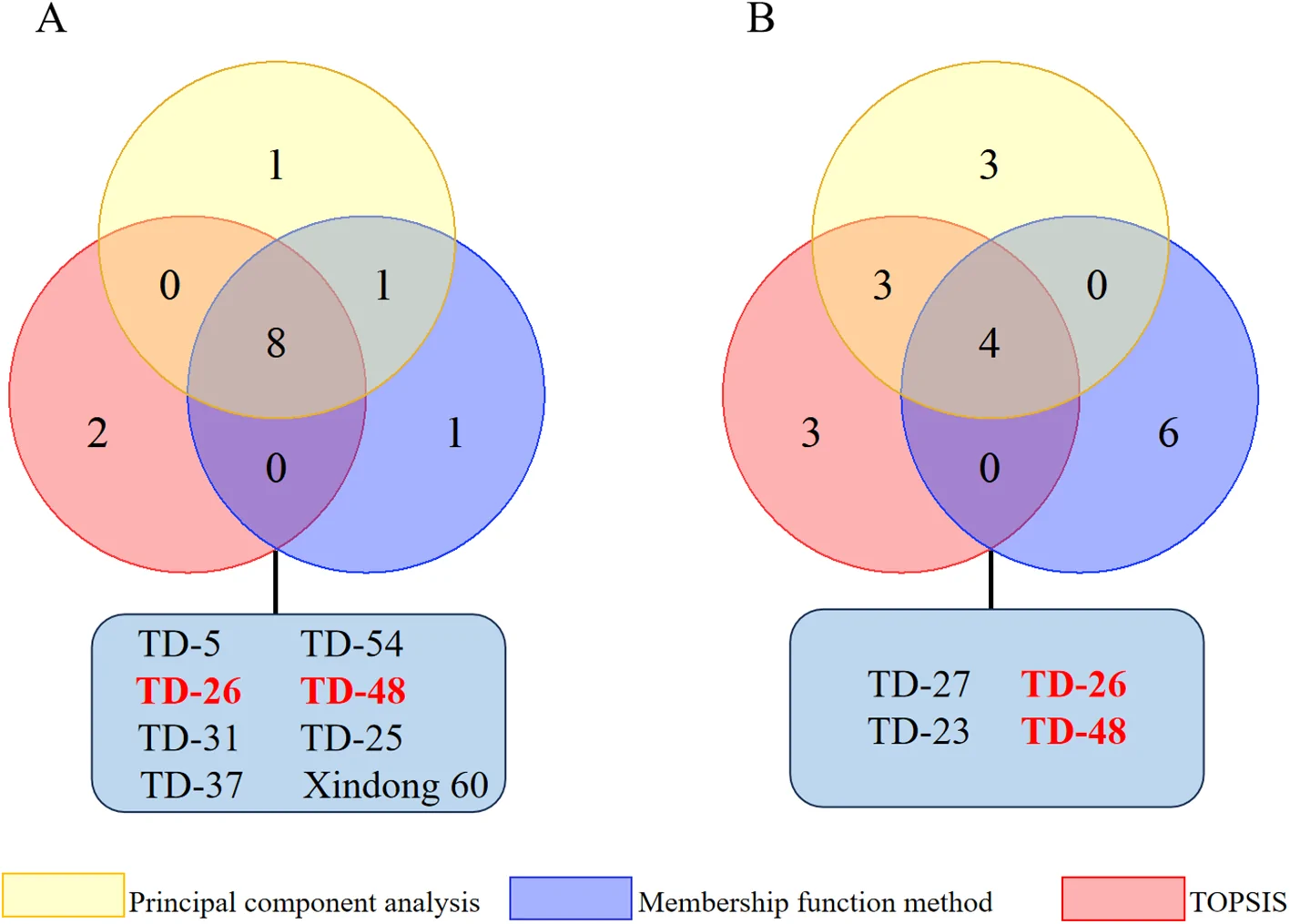
Screening results of yield-related traits of 52 advanced wheat lines based on a PCA-Membership Function-TOPSIS Collaborative System. **(A)** Venn diagram of the top ten high-yielding lines identified by three comprehensive evaluation methods in 2023; **(B)** Corresponding Venn diagram for the top ten lines identified in 2024. Yellow, blue, and red represent lines selected based on the PCA, membership function, and TOPSIS, respectively. Red lines within the blue box indicate elite lines that were consistently identified by all three evaluation methods.

To further confirm the reliability of core candidate line screening, this study adopted a joint validation strategy by integrating PCA, Membership Function Method (D) and TOPSIS (F), and visualized the intersection of top 10 candidate lines selected in 2023 and 2024 via Venn diagram. As shown in **Figure 5**, the common intersection of the three methods in 2023 contained 8 core lines: TD-5, TD-54, TD-26, TD-48, TD-31, TD-25, TD-37 and Xindong 60, and the pairwise intersections of PCA & TOPSIS, and Membership Function Method & TOPSIS each contained 9, 8 and 9 lines. In 2024, the common intersection of the tri-method contained 4 core lines: TD-26,TD-23,TD-27 and TD-48, while the intersection of PCA and TOPSIS contained 7 core line, and the intersection of Membership Function Method and TOPSIS contained 4 core lines (**Figure 5B**). Notably, TD-26 and TD-48 were stably ranked among the top 10 in all three evaluation systems across two consecutive years; combined with the clustering results, the two lines were classified as the “High-yield and inter-annually consistent type” (**Figure 3**). The above multi-dimensional joint validation confirmed that the two lines exhibited inter-annually consistent and superior comprehensive performance in yield-related traits, identifying them as core elite lines for high-yield and stable-yield breeding with substantial application potential.

## 4 Discussion

This study integrated three multi-criteria decision-making methods, namely PCA, membership function method, and TOPSIS, to conduct a comprehensive cross-year and multi-trait comprehensive evaluation of 52 advanced wheat lines in Southern Xinjiang. The results showed that the two lines, TD-26 and TD-48, consistently ranked among the top ten in both 2023 and 2024. They demonstrated excellent high-yield potential and inter-annual consistency, indicating that they were less affected by environmental fluctuations and had good wide adaptability. The stability of these elite lines was further corroborated by the joint evaluation of mean D-values and their coefficients of variation (CV), as illustrated in **Supplementary Figure 3**. Notably, TD-26 and TD-48 occupied the optimal “high meanlow CV” quadrant, confirming their superior and stable performance across growing seasons. Compared to single-model approaches, this screening strategy based on multi-model consensus can effectively mitigates evaluation bias inherent in individual models and significantly, thereby significantly improving the reliability and reproducibility of germplasm selection (Yan and Kang, 2002; Olivoto et al., 2019a). In crop breeding practice, the genotype-environment interaction (G×E) is a pivotal factor that restricts the breeding efficiency and affects the promotion potential of lines (Kang, 2020). Through multiple-point field trials over two years conducted on the same set of materials, this study demonstrated that relying solely on single-year phenotypic data often leads to the misidentification or omission of elite lines due to inter-annual environmental fluctuations. This challenge is particularly acute in arid and semi-arid regions, where inter-annual environmental fluctuations significantly affect the agronomic performance (Al-Sayaydeh et al., 2023; Mullualem et al., 2024). Previously, in the evaluation of hard red wheat in northwestern Ethiopia, the contribution rate of the environmental effect reached 40.53%, and the contribution rate of the G×E interaction was as high as 43.78%, while the contribution rate of the genotype effect itself was only 15.69% (Menzir et al., 2025). These findings underscore the critical need for multi-environmental and cross-annual comprehensive evaluation in wheat variety screening. Through the joint validation of three methods, this study not only identified lines with outstanding high-yield potential within the evaluation year but, more importantly, discovered elite lines maintaining sustained superiority across different growing seasons (Alemayehu et al., 2025). Notably, although the three evaluation methods exhibited high concordance in identifying the top 10 lines, there were still differences. These disparities reflect the varying sensitivities of each model to trait weighting, suggesting that multi-method integration provides a more holistic evaluation of line performance (Yan et al., 2007). Similarly, the study found that the stability screening results of the Additive Main Effects and Multiplicative Interaction (AMMI) model did not fully align with those of the Multi - trait Stability Index (MTSI). The AMMI model tends to select genotypes with stable yields across different environments, while the MTSI focuses more on the comprehensive stability of the overall performance of multiple agronomic traits (Olivoto et al., 2019b). Through multi-dimensional joint verification, this study identified core breeding lines that consistently performed well across all evaluation systems. These selected materials exhibited broad adaptability and inter-annual consistency, preliminarily fulfilling the current breeding objectives for “high yield, stable performance, and wide adaptability” required for optimal cultivars in the arid regions of southern Xinjiang at present.

### 4.1 Key yield-related traits contribution and prediction model construction

Through a stepwise regression analysis using the comprehensive evaluation value of the subordinate function as the dependent variable, this study established a mathematical model for predicting the yield potential of breeding materials: D = 0.16 + 0.294GY+0.123GWS+0.064TKW+0.107GNP+0.054SNP+0.018BSSN (R^2^=0.983, P<0.001). The regression model exhibited a high coefficient of determination (R^2^= 0.983), indicating that the six key traits (GY, GWS, TKW, GNP, SNP, and BSSN) included are sufficient to explain 98.3% of the variation in the comprehensive evaluation value. This confirms that the above-mentioned traits can highly summarize the comprehensive performance of different wheat lines, and thus can serve as core evaluation indicators for the rapid screening of advanced lines. Based on this simplified strategy, not only can the workload of large-scale line screening be effectively reduced, but also the breeding efficiency can be significantly improved.

The standardized coefficients (Beta) derived from the final optimized model reflected the relative weight of each trait on the comprehensive evaluation. Among the retained traits, GY exhibited the highest Beta value (0.684), indicating its dominant influence. GNP (0.281) and GWS (0.276) ranked second and third, reflecting their substantial secondary roles (**Supplementary Table 7**). The dominant influence of GY, along with the complementary roles of GNP and GWS, aligns with the coarse-fine tuning theory of yield components proposed by predecessors, which emphasizes the hierarchical and compensatory regulation of grain number (coarse tuning) and grain weight (fine tuning) in determining final yield (Slafer et al., 2014). The substantial contributions of GWS and GNP suggest that both sink size (grain number) and sink strength (grain weight) are critical for achieving high comprehensive performance, a conclusion well supported by the physiological framework of sink capacity proposed by predecessors (Alonso et al., 2018). From a physiological breeding perspective, the high contribution rates of GWS and GWP underscore the central role of source-sink coordination in achieving high wheat yields. Specifically, GWS characterizes the storage capacity of the spike, whereas GWP reflects the plant’s capacity for photoassimilate production and distribution. In addition, although SNP and TKW exhibited a weak phenotypic correlation that shifted from slightly positive in 2023 (r≈0.11, **Figure 2A**) to slightly negative in 2024 (r≈-0.04, **Figure 2B**), their positive regression coefficients (SNP = 0.054; TKW = 0.064) suggest that this apparent trade-off can be overcome. Within an optimized genetic background—potentially via judicious selection of combining ability and the stabilization of genotype-by-environment interactions—these traits can achieve synergistic improvement. This aligns with the physiology of elite cultivars, which often avoid the typical yield component antagonism (i.e., increased grain number decreasing grain weight) by improving the balance between assimilate accumulation (source) and the expansion of an effective filling sink (more spikelets and higher TKW) (Zhao et al., 2023; Abbai et al., 2024). Consequently, the positive contributions of SNP and TKW provide empirical evidence for breaking the yield component trade-off in wheat breeding. Furthermore, the retention of GY as a composite output indicator (regression coefficient=0.294), reinforces that multi-trait evaluation captures the true potential of elite lines more comprehensively than selection based solely on yield. Overall, our six-trait regression model not only provides a low-cost, high-efficiency screening tool for wheat breeding, but also quantitatively validates that “strong spike sink, high productivity per plant, and source-sink balance” constitute the common characteristics of high-yield wheat cultivars (Zhang et al., 2025).

### 4.2 Methodological significance and application prospects

Based on the multi-dimensional elite wheat screening system of “multi-method comprehensive evaluation, key trait prediction, and inter-annually consistent validation” established in this study, we put forward the following insights for wheat breeding and practical application in the arid region: (1) Under the background of variable climates, the performance of wheat cultivars is significantly affected by genotype-environment interaction (G×E) (**Supplementary Figures 1**, **2**). The joint verification strategy of PCA-membership function-TOPSIS can effectively make up for the limitations inherent in single evaluation methods. This consensus-based framework offers a more comprehensive perspective for identifying high-yield and inter-annually consistent cultivars, and may reduce the risk of method-dependent bias in cultivar selection. (2) The six-trait regression model developed herein (including GY, GWS, TKW, GNP, SNP, and BSSN) explained 98.3% of the variance in comprehensive performance. This provides a highly efficient and cost-effective simplified index system for the large-scale, and rapid screening of advanced lines. (3) Based on inter-annual consistency tracking, two wheat lines,TD-48 and TD-26, have consistently shown excellent performance. They can be directly used as core elite parents in the wheat-growing areas of southern Xinjiang, or recommended for the next-step regional trials for production verification. (4) In recent years, multiple studies highlight that in future breeding practices, attention should be paid to the coordinated improvement of spikelet number and grain weight to optimize yield architecture and alleviate the yield trade-off (Zhao et al., 2023; Abbai et al., 2024). The results of the trait correlation analysis in this study validate this trajectory (**Figure 2**).

While the PCA-Membership Function-TOPSIS collaborative system offers a robust multi-trait integration framework that effectively identifies top-performing lines with consistent inter-annual performance, we acknowledge its complementary role alongside dedicated models (such as AMMI, GGE). Subsequent breeding work should further incorporate stability parameters into the comprehensive evaluation system to develop cultivars with both high-yield potential and climate adaptability (Han et al., 2025b). Given that climate change is driving significant shifts in global wheat suitability areas (Alemayehu et al., 2024), there is an urgent need for forward-looking breeding strategies that prioritize regional adaptation.

## 5 Conclusion

This study integrated PCA, membership function and TOPSIS to construct a multi-model comprehensive evaluation system. Combined with stepwise regression, it systematically evaluated the yield potential and inter-annual consistency of 52 advanced wheat lines. The key conclusions are as follows: (1) TD-48 and TD-26 consistently ranked among the top ten in the comprehensive performance in both 2023 and 2024, demonstrating excellent high-yield and inter-annually consistent characteristics, and can be regarded as key materials for breeding in arid regions. (2) The prediction model constructed by stepwise regression (D = 0.16 + 0.294GY+0.123GWS+0.064TKW+0.107GNP+0.054SNP+0.018BSSN (R^2^=0.983, P<0.001). indicates that only six traits can explain 98.3% of the phenotypic variation, providing simplified key indicators for the selection of advanced lines. The collaborative system established here provides a replicable methodological reference for wheat breeding in arid ecosystems, and the elite lines identified serve as valuable foundational germplasm resources for cultivating high-yield and stable-yield wheat cultivars.

## Data Availability

The original contributions presented in the study are included in the article/**Supplementary Material**. Further inquiries can be directed to the corresponding authors.
