# Supplementary material for: Joint validation of yield and inter-annual consistency and elite lines selection in 52 advanced wheat lines based on a PCA-membership function-TOPSIS collaborative system
Source: Front Plant Sci. 2026 Aug 25;17:1907156. doi: 10.3389/fpls.2026.1907156 (PMC13550142; doi:10.3389/fpls.2026.1907156)
Supplement: Supplementary file 1 [file DataSheet1.docx]

Supplementary Material

# 1 Supplementary Figures

**Supplementary Figure 1**. BSSN and its Frequency distribution in 2023 and 2024. (A) The BSSN distribution in 2023 (red) and 2024 (blue). BSSN, basal sterile spikelet number. Key statistics (mean; standard deviation, SD; coefficient of variation, CV; and sample size n) are annotated for each year. Jittered points represent individual observations. (B) The frequency distribution of BSSN for 2023 (red) and 2024 (blue) overlaid. Dashed lines indicate the mean value for each year (red=2023 mean; blue=2024 mean).

Note: The exceptionally high CV in 2023 (81.6%) reflects greater dispersion (including low-value outliers) compared to 2024 (61.4%).

**Supplementary Figure 2.** Key meteorological conditions during the wheat grain-filling period in 2024 and 2025. (A) Daily temperature dynamics in 2024, showing mean temperature (Tmean, orange line), maximum (Tmax, red line), minimum (Tmin, blue line), and daily range (shaded area); (B) Daily precipitation in 2024—total precipitation is 30.9 mm; (C) Daily temperature dynamics in 2025, with same legend as (A); (D) Daily precipitation in 2025—no precipitation recorded during the entire period.Note: Years in the original manuscript denote sowing years (2023, 2024); grain filling shown here occurred in 2024 and 2025.

# Supplementary Figure 3. Relationship between mean D‑value and coefficient of variation (CV) across advanced wheat lines. The small red dots in the figure highlight the two advanced lines (TD-26 and TD-48) as well as the check cultivar (Xindong 60). The remaining advanced wheat lines are represented by small black dots. The CV reflects the relative variability within each genotype under the tested conditions. Statistical details are provided in Supplementary Table 1.

# 2 Supplementary Tables

**Supplementary Table 1** Abbreviations used in this study.

| Abbreviation | English meaning |
| --- | --- |
| SL | Spike Length |
| SNP | Spike number per plant |
| SNS | Spikelet number per spike |
| BSSN | Basal sterile spikelet number |
| GNS | Grain number per spike |
| GWS | Grain weight per spike |
| GWP | Grain weight per plant |
| TKW | Thousand kernel weight |
| GNP | Grain number per plant |
| GY | Grain yield |

**Supplementary Table 2** Descriptive statistics and variation analysis for yield-related traits of 52 advanced wheat lines across two growing seasons.

| Traits | 2023 | | | | | 2024 | | | | |
| --- | --- | --- | --- | --- | --- | --- | --- | --- | --- | --- |
|  | Mean | SD | Min | Max | CV (%) | Mean | SD | Min | Max | CV (%) |
| SL | 7.63 | 0.89 | 5.62 | 9.80 | 11.69 | 7.90 | 1.02 | 5.52 | 10.28 | 12.86 |
| SNP | 3.19 | 0.50 | 2.00 | 4.00 | 15.68 | 2.29 | 0.55 | 1.00 | 3.60 | 24.01 |
| SNS | 16.92 | 1.71 | 13.20 | 20.20 | 10.13 | 16.40 | 1.54 | 12.00 | 19.40 | 9.42 |
| BSSN | 1.18 | 0.96 | 0.00 | 2.80 | 81.57 | 1.12 | 0.69 | 0.00 | 2.20 | 61.40 |
| GNS | 31.94 | 8.77 | 18.60 | 55.40 | 27.45 | 44.26 | 8.35 | 30.20 | 68.40 | 18.86 |
| GWS (g) | 1.59 | 0.52 | 0.81 | 2.91 | 32.57 | 2.28 | 0.46 | 1.19 | 3.63 | 20.22 |
| GWP (g) | 4.53 | 1.93 | 1.99 | 8.52 | 42.61 | 3.84 | 1.13 | 1.91 | 7.04 | 29.31 |
| TKW (g) | 48.23 | 5.24 | 36.69 | 59.78 | 10.87 | 45.05 | 4.72 | 35.39 | 60.22 | 10.47 |
| GNP | 95.71 | 38.92 | 38.00 | 185.60 | 40.66 | 79.55 | 25.62 | 35.00 | 157.00 | 32.21 |
| GY (t/ha) | 8.19 | 1.25 | 5.51 | 10.21 | 15.30 | 8.01 | 0.85 | 5.47 | 10.01 | 10.66 |

**Supplementary Table 3** Sensitivity analysis of BSSN coefficient of variation (CV) based on entry means across two years.

| Year | Treatment / Scenario | N | Mean | SD | CV (%) |
| --- | --- | --- | --- | --- | --- |
| 2023 | Original raw data (Control) | 52 | 1.18 | 0.96 | 81.57 |
|  | Excluding zero-inflated lines (BSSN ≤ 0.5) | 35 | 1.68 | 0.76 | 45.09 |
|  | Excluding extreme high-value lines (BSSN > 2.0) | 38 | 0.71 | 0.62 | 88.3 |
| 2024 | Original raw data (Control) | 52 | 1.12 | 0.69 | 61.4 |
|  | Excluding zero-inflated lines (BSSN ≤ 0.5) | 39 | 1.42 | 0.53 | 37.3 |
|  | Excluding extreme high-value lines (BSSN > 2.5) | 43 | 0.92 | 0.58 | 62.72 |

**Supplementary Table 4** Estimates of broad-sense heritability (*H²*) for key agronomic traits across years and within individual years (2023 and 2024).

| Trait | *H²* across-years | 2023 | 2024 |
| --- | --- | --- | --- |
| SNP | 0.498 | 0.540 | 0.824 |
| SL | 0.955 | 0.942 | 0.872 |
| SNS | 0.896 | 0.880 | 0.768 |
| BSSN | 0.879 | 0.908 | 0.753 |
| GNS | 0.885 | 0.895 | 0.705 |
| GWS | 0.881 | 0.863 | 0.632 |
| GWP | 0.872 | 0.936 | 0.798 |
| TKW | 0.957 | 0.939 | 0.965 |
| GNP | 0.872 | 0.936 | 0.798 |
| GY | 0.884 | 0.988 | 0.978 |

Note: This table reports broad-sense heritability (*H²*) estimates for each trait calculated (i) across the two-year dataset (2023–2024) and (ii) separately within each year (2023 and 2024). Estimates are based on 52 genotypes evaluated each year in a RCBD. The across-year *H²* values summarize the consistency of genetic differences among genotypes over years by accounting for genotype×year interaction and residual variation, while the within-year H² values reflect genetic repeatability within a single year under that year’s environmental conditions. Variance components were derived from the corresponding ANOVA/mixed-model partitioning of variation. The abbreviation of phenotypic traits is shown in Table S1.

**Supplementary** Table 5 Statistical validation of comprehensive evaluation indices in 2023 and 2024.

| Year | Index | Normality (Shapiro-Wilk) | Homogeneity (Levene) | Selected Method | Test Statistic | P-value |
| --- | --- | --- | --- | --- | --- | --- |
| 2023 | Membership Function  D-value | W=0.992, P=0.173 | P<0.001 | Welch ANOVA | F_51,71.946_=55.678 | <0.001 |
|  | Principal component comprehensive score | W=0.989, P=0.056 | P<0.001 | Welch ANOVA | F_51,71.984_=52.412 | <0.001 |
|  | TOPSIS C | W=0.987, P=0.016 | P<0.001 | Kruskal-Wallis H | H=136.913 | <0.001 |
| 2024 | Membership Function  D-value | W=0.993, P=0.279 | P＜0.001 | Welch ANOVA | F_51,71.998_=36.475 | <0.001 |
|  | Principal component comprehensive score | W=0.994, P=0.434 | P<0.001 | Welch ANOVA | F_51,72.014_=6.284 | <0.001 |
|  | TOPSIS C | W=0.974, P<0.001 | P<0.001 | Kruskal-Wallis H | H=156.460 | <0.001 |

**Supplementary Table 6** Two-year average values of the membership function D of 52 advanced wheat lines.

| Line | D Value | Ranking | Line | D Value | Ranking |
| --- | --- | --- | --- | --- | --- |
| TD-1 | 0.29 | 50 | TD-26 | 0.76 | 1 |
| TD-2 | 0.39 | 35 | TD-27 | 0.57 | 16 |
| TD-3 | 0.36 | 40 | TD-28 | 0.51 | 24 |
| TD-4 | 0.37 | 38 | TD-29 | 0.57 | 14 |
| TD-5 | 0.68 | 4 | TD-30 | 0.35 | 43 |
| TD-6 | 0.40 | 34 | TD-31 | 0.59 | 11 |
| TD-7 | 0.36 | 42 | TD-32 | 0.67 | 6 |
| TD-8 | 0.47 | 28 | TD-33 | 0.39 | 36 |
| TD-9 | 0.58 | 13 | TD-34 | 0.56 | 18 |
| TD-10 | 0.36 | 41 | TD-35 | 0.60 | 10 |
| TD-11 | 0.49 | 25 | TD-36 | 0.57 | 15 |
| TD-12 | 0.38 | 37 | TD-37 | 0.75 | 2 |
| TD-13 | 0.44 | 30 | TD-38 | 0.31 | 49 |
| TD-14 | 0.53 | 22 | TD-39 | 0.42 | 32 |
| TD-15 | 0.34 | 46 | TD-40 | 0.41 | 33 |
| TD-16 | 0.42 | 31 | TD-41 | 0.48 | 26 |
| TD-17 | 0.58 | 12 | TD-42 | 0.35 | 45 |
| TD-18（R） | 0.35 | 44 | TD-43 | 0.57 | 17 |
| TD-18（W） | 0.52 | 23 | TD-44 | 0.47 | 27 |
| TD-19 | 0.27 | 51 | TD-45 | 0.54 | 20 |
| TD-20 | 0.53 | 21 | TD-46 | 0.34 | 47 |
| TD-21 | 0.18 | 52 | TD-48 | 0.70 | 3 |
| TD-22 | 0.44 | 29 | TD-51 | 0.33 | 48 |
| TD-23 | 0.62 | 7 | TD-52 | 0.36 | 39 |
| TD-24 | 0.55 | 19 | TD-54 | 0.68 | 5 |
| TD-25 | 0.60 | 9 | Xindong 60 | 0.61 | 8 |

**Supplementary Table 7** Results of the stepwise linear regression analysis.

| Variable | Non-standardized  coefficients | | Standardized coefficients | | | Collinearity  statistics | |
| --- | --- | --- | --- | --- | --- | --- | --- |
|  | B | Standardized  error | Beta | t | Sig. | Tolerance | VIF |
| Constant | 0.16 | 0.010 | - | 16.748 | 0.000*** | - | - |
| GY | 0.294 | 0.009 | 0.684 | 33.242 | 0.000*** | 0.885 | 1.129 |
| GWS | 0.123 | 0.011 | 0.276 | 11.436 | 0.000*** | 0.644 | 1.553 |
| TKW | 0.064 | 0.010 | 0.133 | 6.518 | 0.000*** | 0.905 | 1.104 |
| GNP | 0.107 | 0.012 | 0.281 | 8.85 | 0.000*** | 0.371 | 2.692 |
| SNP | 0.054 | 0.011 | 0.127 | 4.873 | 0.000*** | 0.551 | 1.815 |
| BSSN | 0.018 | 0.007 | 0.060 | 2.606 | 0.0130* | 0.710 | 1.409 |
| R^2^ | 0.983 | | | | | | |
| Adj.R^2^ | 0.981 | | | | | | |
| F | F=437.192,***p<0.001 | | | | | | |

Note: GY, grain yield (t/ha); GWS, grain weight per spike (g); TKW, thousand kernel weight (g); GNP, grain number per plant; SNP, spike number per plant; BSSN, basal sterile spikelet number.

**p* < 0.05, ***p* < 0.01, ****p* < 0.001.
